# Stimulation of RAS-dependent ROS signaling extends longevity by modulating a developmental program of global gene expression

**DOI:** 10.1126/sciadv.adc9851

**Published:** 2022-11-30

**Authors:** Robyn Branicky, Ying Wang, Arman Khaki, Ju-Ling Liu, Maximilian Kramer-Drauberg, Siegfried Hekimi

**Affiliations:** Department of Biology, McGill University, Montreal, Quebec H3A 1B1, Canada.

## Abstract

We show that elevation of mitochondrial superoxide generation increases *Caenorhabditis elegans* life span by enhancing a RAS-dependent ROS (reactive oxygen species) signaling pathway (RDRS) that controls the expression of half of the genome as well as animal composition and physiology. RDRS stimulation mimics a program of change in gene expression that is normally observed at the end of postembryonic development. We further show that RDRS is regulated by negative feedback from the superoxide dismutase 1 (SOD-1)-dependent conversion of superoxide into cytoplasmic hydrogen peroxide, which, in turn, acts on a redox-sensitive cysteine (C118) of RAS. Preventing C118 oxidation by replacement with serine, or mimicking oxidation by replacement with aspartic acid, leads to opposite changes in the expression of the same large set of genes that is affected when RDRS is stimulated by mitochondrial superoxide. The identities of these genes suggest that stimulation of the pathway extends life span by boosting turnover and repair while moderating damage from metabolic activity.

## Teaser

High ROS generation by the oncogene RAS promotes longevity through changes of gene expression that boost turnover and repair.

## INTRODUCTION

Reactive oxygen species (ROS) can potentially damage macromolecules, which is why the oxidative stress theory of aging proposes that damage from ROS is a main cause of aging. However, since at least 2007 (*1*), a variety of studies have yielded the interpretation that elevated mitochondrial ROS (mtROS) might be able to increase longevity (*2*–*8*). The simplest way to robustly induce ROS-dependent longevity is via treatment with the mitochondrial superoxide generator paraquat (PQ), which increases life span at low doses with minimal effects on other phenotypes (*6*, *7*). PQ increases life span even when treatment is applied only during adulthood (*6*). Thus, worm physiology is continuously sensitive to PQ, not only during particular developmental stages.

Numerous enzymatic systems control ROS generation and removal. This allows ROS to function as signaling molecules that modulate and act as relays in signal transduction cascades (*9*–*11*). It is by their effect on signaling, particularly their action on redox-sensitive cysteines (*12*–*14*), that increased ROS levels might activate protective mechanisms and increase longevity (*8*, *15*–*18*). The conserved NRF2/*skn-1* pathway is likely the most extensively studied pathway capable of transforming ROS signals into increased survival (*19*).

Many studies have sought to document changes in gene expression associated with interventions that increase life span. In particular, the attempt has been made to identify core aging processes by finding gene expression changes that are common across interventions (mutations, drugs, and diet) or across species (*17*, *20*–*29*). However, no common gene set or common mechanism has yet been identified in this way.

The study of long-lived mutants has documented a requirement for stress response pathways to license longevity. In particular, the life-span extension that results from dysfunctional mitochondria often requires intact mitochondrial stress responses (*17*, *18*, *30*–*33*). However, here too, the analysis of gene expression changes accompanying mitochondrial dysfunction and/or elevated oxidative stress has so far failed to show any consensus pattern (*17*, *27*, *34*).

The small guanosine triphosphatase (GTPase) RAS is involved in many signal transduction pathways that regulate development and growth and has been implicated in aging (*35*–*37*). RAS activation leads to the production of ROS by a variety of mechanisms (*38*–*44*). In return, the RAS pathway is itself regulated by ROS in mammals (*45*) and in *Caenorhabditis elegans* (*46*, *47*). A redox-sensitive cysteine (C118) of the RAS protein itself can be directly oxidized by nitric oxide, superoxide, and hydrogen peroxide (H_2_O_2_) (*48*–*50*).

A powerful tool to study the roles of redox-sensitive cysteines is by replacement with a serine that cannot be oxidized (*13*, *51*–*53*) or by an aspartic acid to mimic oxidation (fig. S1) (*52*–*54*). The development of the hermaphrodite vulva of *C. elegans* is controlled by a signaling cascade that includes the Kras homolog LET-60ras and is regulated by oxidation of C118 (*47*). C118 oxidation by PQ or replacement of C118 by aspartic acid (C118D) suppresses LET-60ras signaling and vulva formation, while replacement of C118 by serine (C118S) enhances RAS signaling and vulva formation. Although PQ generates superoxide, oxidation of C118 required cytoplasmic H_2_O_2_ generation, as it can be prevented by *N*-acetyl-cysteine (NAC) treatment and requires the presence of the cytoplasmic SOD-1 (*47*).

Here, we show the following: (i) Mitochondrial sources of superoxide, including following PQ treatment, act on longevity by increasing cytoplasmic H_2_O_2_ generation. (ii) Oxidation of cysteine C118 of LET-60ras by H_2_O_2_ is necessary for life-span extension triggered by PQ treatment. (iii) PQ treatment at the prolongevity dosage of 0.1 mM affects the expression of thousands of genes, up-regulating genes associated with the high-quality synthesis of cellular constituents and down-regulating genes necessary for intermediary metabolism and energy generation. (iv) The action of PQ modulates a program of gene expression associated with the end of postembryonic development. (v) The C118S and C118D mutations of LET-60ras produce changes in gene expression that, respectively, are similar to those produced by PQ treatment (C118S) or are the reverse (C118D). By reverse, we mean that the genes that are up-regulated by PQ are down-regulated and the genes that are down-regulated are up-regulated. This points to a global regulatory function of C118 oxidation and RAS-dependent ROS signaling (RDRS). (vi) The C118S mutation renders the expression of most genes refractory to PQ treatment, while treatment of C118D with PQ completely reverses the effect of C118D on gene expression. That is, the gene expression in C118D mutants treated with PQ is mostly like that of wild type (WT) treated with PQ. (vii) PQ affects body composition and metabolic parameters in ways that are consistent with the observed patterns of gene expression. (viii) Comparison of the pattern of gene expression produced by PQ with published patterns associated with longevity allowed us to identify a previously elusive prolongevity gene expression signature. (ix) Our findings allowed us to formulate a model in which PQ acts downstream of LET-60ras to enhance RDRS.

## RESULTS

### Mitochondrial sources of superoxide act on longevity by increasing cytoplasmic H_2_O_2_ generation

**Fig. 1.**
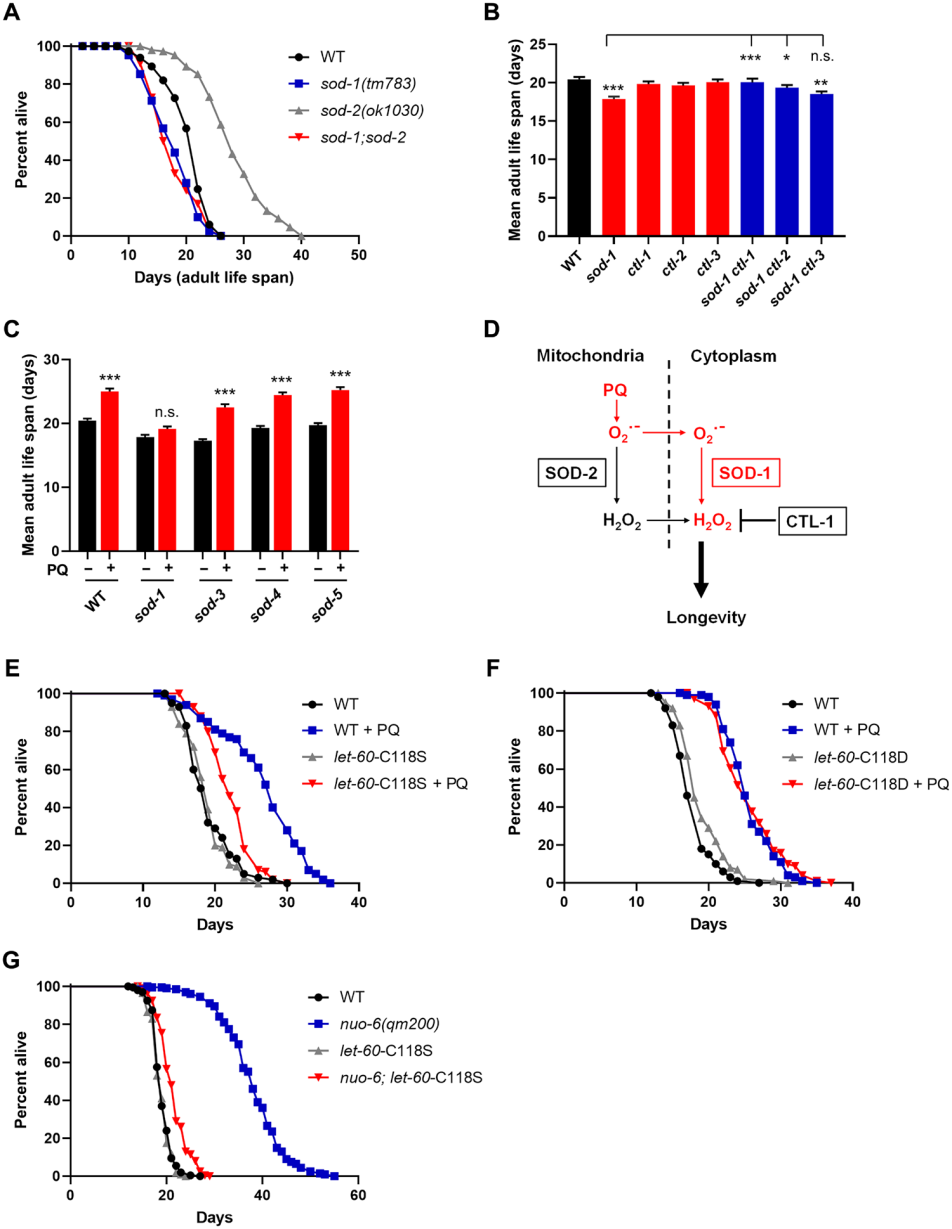
Mitochondrial superoxide increases longevity via cytoplasmic H_2_O_2_ and requires oxidation of cysteine 118 of LET-60. (**A**) Life spans of *sod-1(tm783)* and *sod-2(ok1030)* single and double mutants. (**B**) Life spans of *sod-1(tm783)*, *ctl-1(ok1242)*, *ctl-2(ok1137)*, and *ctl-3(ok2042)* single and double mutants. All single mutants are compared to the wild type (WT), and only significant differences are indicated; double mutants are compared to *sod-1*. (**C**) Life spans of *sod-1(tm783)*, *sod-3(tm760)*, *sod-4(gk101)*, and *sod-5(tm1146)* with and without treatment with 0.1 mM paraquat (PQ) from hatching. Comparisons are made for each genotype with and without PQ treatments. (**D**) Model of reactive oxygen species (ROS), enzymes, and compartments. (**E**) Life span of *let-60-*C118S mutants treated with 0.1 mM PQ from hatching. (**F**) Life span of *let-60-*C118D mutants treated with 0.1 mM PQ from hatching. (**G**) Life span of *nuo-6(qm200); let-60*-C118S double mutants. ***P < 0.001; **P < 0.01; *P < 0.05. n.s., not significant. Bars represent means and error bars represent SEM. All numerical values and statistics are presented in table S1.

SODs are the only enzymes that directly lower superoxide levels by converting it to H_2_O_2_. We confirm that loss of the main mitochondrial SOD (SOD-2) lengthens adult life span (*55*) but find that the loss of the main cytoplasmic SOD (SOD-1) shortens adult life span (Fig. 1A and table S1). These effects are not additive. Rather, the loss of SOD-1 completely suppresses the prolongevity effect of the loss of SOD-2 (Fig. 1A). This suggests that the elevated mitochondrial superoxide resulting from the loss of SOD-2 needs to be converted to H_2_O_2_ to act on longevity. This conclusion is reinforced by observations with mutations in catalases, which remove H_2_O_2_ by converting it to water and oxygen. In *C. elegans*, the two major catalases are CTL-1, which is cytoplasmic, and CTL-2, which is peroxisomal, like vertebrate catalase (*56*, *57*). Although the loss of any one of the catalases does not affect life span by itself, the loss of CTL-1 or CTL-2 suppressed the short life span due to loss of SOD-1 (Fig. 1B), presumably by allowing for a level of cytoplasmic H_2_O_2_ sufficient for WT longevity. The fact that the loss of CTLs has no effect on life span by itself suggests that SOD-1 always produces sufficient H_2_O_2_ for WT longevity.

PQ is believed to act mainly at mitochondrial complex I (*58*, *59*). We have developed a protocol by which treatment of the WT with 0.1 mM PQ markedly increases life span (*6*). This effect is fully suppressed by loss of SOD-1, but not SOD-3, SOD-4, or SOD-5 (Fig. 1C). Treatment of *sod-2* mutants with PQ could not be scored, as it leads to developmental arrest (*8*). Thus, SOD-1 is required for the prolongevity action of both the loss of SOD-2 (Fig. 1A) and PQ treatment (Fig. 1C). Together, the observations in this section lead to a model that suggests that superoxide uses specialized channels to exit the mitochondria matrix (*60*, *61*) and is then converted into prolongevity H_2_O_2_ by cytoplasmic SOD-1 and removed, at least in part, by catalases (Fig. 1D).

### Oxidation of cysteine C118 of LET-60ras by cytoplasmic H_2_O_2_ is necessary for life-span extension by PQ

In a previous study, PQ and the loss of SOD-1 were shown to affect vulva formation by affecting the oxidation of the C118 cysteine of the gain-of-function (*n1046*gf) LET-60ras allele (*47*). A crucial result of this study was that replacement of C118 by serine (C118S), which cannot be oxidized by H_2_O_2_, enhances LET-60ras signaling, while replacement by aspartic acid (C118D), which mimics constitutive cysteine oxidation, inhibits signaling. Here, we used the same C118 mutations, but in the *let-60ras* WT background, to study the mechanisms of life-span extension by PQ. These mutants will often be referred to simply as C118S and C118D.

Neither C118S nor C118D mutations have effects on life span by themselves (Fig. 1, E and F). However, while C118S strongly prevents the life span–extending effect of PQ (Fig. 1E), PQ still extends the life span of C118D to the same degree as it does that of WT (Fig. 1F). This means that PQ does not extend life span by oxidizing C118. Rather, C118 oxidation (or mimicking its oxidation by replacement with aspartic acid) is required for life-span extension by PQ.

### The prolongevity ROS from *nuo-6(qm200)* act through C118

As shown above, LET-60ras-C118 is sensitive to cytoplasmic H_2_O_2_ as indicated by the roles of SOD-1 and CTL-1. However, other aspects of the data (Fig. 1A) and our previous findings (*47*) suggested a model in which the superoxide that is converted to H_2_O_2_ has its origin in the mitochondria (Fig. 1D). In particular, mitochondrial complex I is almost certainly a main site of superoxide generation by PQ (*58*, *59*). However, PQ could, in principle, also act at other sites, including in the cytoplasm. To investigate whether the PQ-dependent superoxide that ultimately acts through C118 to extend longevity originates from the mitochondria, we tested whether the C118S mutation, which suppresses PQ life-span extension, also suppresses the life-span extension observed with *nuo-6(qm200)* mutants. *nuo-6(qm200)* is a mutation in a matrix subunit of complex I that results in measurably elevated mitochondrial superoxide generation and a markedly extended life span that is completely suppressed by NAC but not additive to the prolongevity effect of PQ (*30*). We constructed *nuo-6(qm200); let-60-*C118S double mutants and found that the extended life span of *nuo-6* was markedly suppressed by the C118S mutation (Fig. 1G). This supports the model that suggests that it is the superoxide initially generated in the mitochondrial matrix by PQ that is ultimately responsible for the oxidation of C118 and the resultant life-span extension.

### PQ treatment at the prolongevity dosage of 0.1 mM produces a shift in global gene expression

**Fig. 2.**
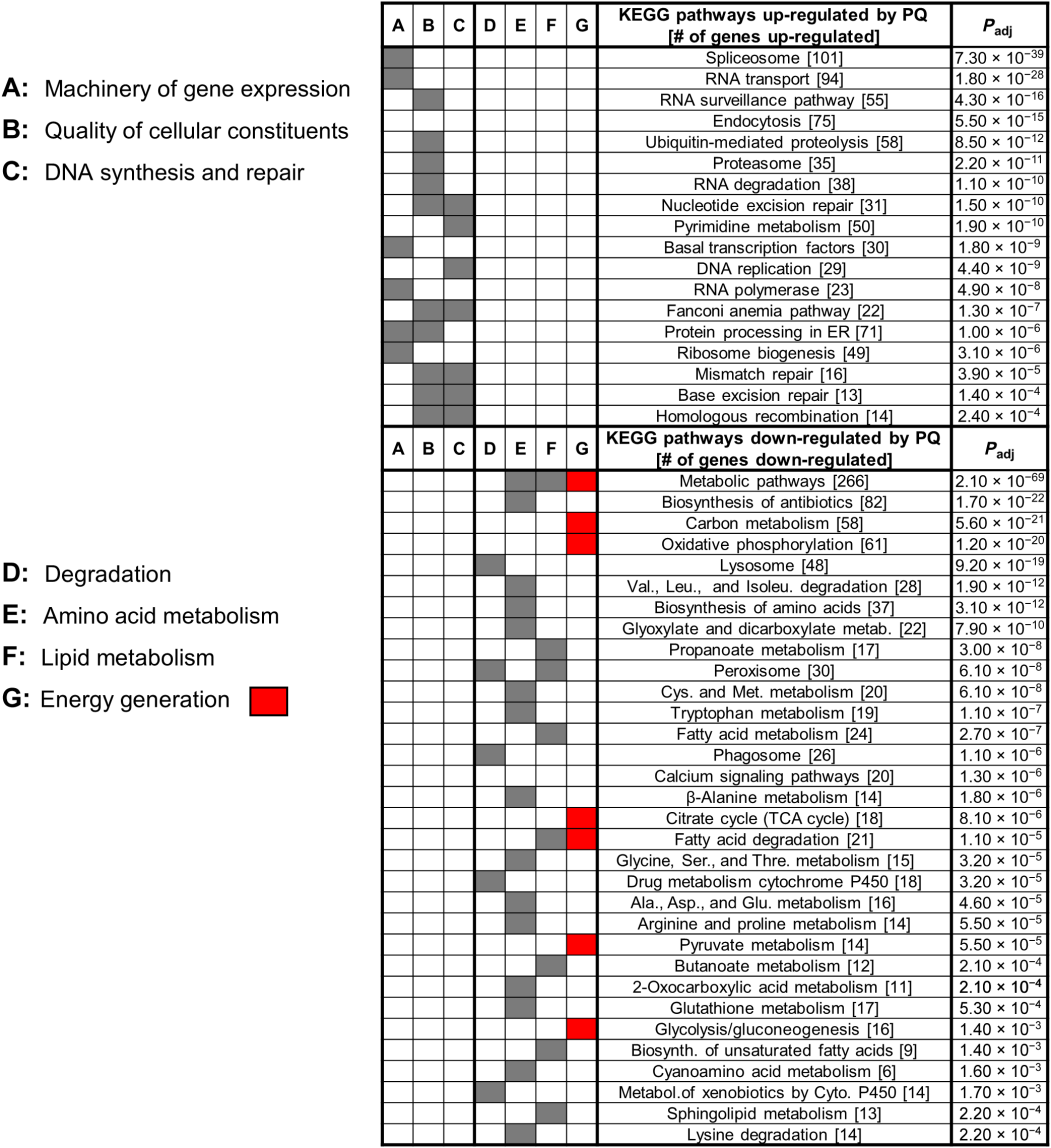
PQ up-regulates genes involved in gene expression, development, growth, and quality control and down-regulates genes involved in degradation, amino acid and lipid metabolism, and energy generation. TCA, tricarboxylic acid. The top Kyoto Encyclopedia of Genes and Genomes (KEGG) pathways enriched in the gene sets that are significantly up- and down-regulated by PQ, ordered by *P* value adjusted for multiple comparisons (*P*_adj_). This figure is a simplification of the complete list of KEGG pathways and biological pathways presented in fig. S3. ER, endoplasmic reticulum; TCA, tricarboxylic acid.

Using RNA sequencing (RNA-seq), we examined the effect of 0.1 mM PQ treatment on gene expression in young adults (YAs) treated from hatching compared to untreated YAs. Throughout the experiments described below, we used a significance cutoff at *P*_adj_ < 0.05. We found that more than half of the genome was affected, with 5618 genes up-regulated by PQ and 5175 genes down-regulated (fig. S2). We used the DAVID Bioinformatics Resources website version 6.8 (david.ncifcrf.gov) for analyses (*62*, *63*). We first analyzed all the genes that could be placed in a Kyoto Encyclopedia of Genes and Genomes (KEGG) pathway (Fig. 2 and fig. S3; Fig. 2 is a simplification of fig. S3 for easier visualization) (*64*). The up-regulated and down-regulated genes were found to be involved in completely different processes. The up-regulated gene set is enriched in 37 KEGG pathways, and the down-regulated gene set is enriched in 56 pathways (fig. S3). Only two weakly enriched pathways (“Wnt signaling” and “inositol phosphate metabolism”) comprise both up- and down-regulated genes. In Fig. 2 and fig. S3, the pathways are ordered as a function of the degree of pathway enrichment. The enrichment in many pathways is extreme, with most genes of the pathway included. Figure S4 provides an illustration using the spliceosome KEGG pathway, in which 101 of the 106 pathway genes are up-regulated by PQ. In some cases, the genes code for proteins that function sequentially in a pathway. Figure S5 provides an illustration for the down-regulated citric acid cycle pathway. In other cases, the pathway corresponds to a defined molecular structure such as the proteasome, with virtually every subunit up-regulated (fig. S6). Other illustrations of marked enrichment or clustering are provided in additional supplementary figures for up-regulated ribosomal biogenesis and mismatch repair pathways (figs. S7 and S8) and for down-regulated glycolysis and gluconeogenesis; peroxisome; and valine, leucine, and isoleucine degradation pathways (figs. S9 to S11).

### Genes up-regulated by PQ are strongly associated with the high-quality synthesis of cellular constituents

To interpret the distribution of up- and down-regulated pathways, we fitted the KEGG pathway into broad categories of biological processes. A clear pattern emerged. Up-regulated genes are involved in all aspects of development and growth. Both DNA synthesis (DNA replication, pyrimidine metabolism, and purine metabolism) and the machinery of gene expression [spliceosome, RNA transport, basal transcription factors, RNA polymerase, protein processing in the endoplasmic reticulum (ER), and ribosome biogenesis] are turned up (Fig. 2). All major pathways of quality control of cellular constituents are turned up (RNA surveillance, ubiquitin-mediated proteolysis, proteasome, RNA degradation, Fanconi anemia, mismatch repair, base excision repair, and homologous recombination) (Fig. 2). Most of the major pathways of signal transduction are also turned up but to a much lesser degree (Foxo, Notch, TGF-β, and mTOR signaling, as well as dorso-ventral axis formations, Wnt, ErbB, Jak-Stat, inositol phosphate, and insulin signaling) (fig. S3). In addition, many aspects of polysaccharide synthesis are up-regulated (syntheses of glycosaminoglycan, heparan sulfate, heparin, amino and nucleotide sugars, chondroitin sulfate, dermatan sulfate, and N-glycans) (fig. S3), but again, much less so than the machinery of gene expression and quality control. Thus, the up-regulated pathways are expected to lead to a higher turnover of DNA, RNA, proteins, and polysaccharides. This, together with the up-regulation of mechanisms of proofreading, turnover, and repair, predicts cellular constituents of higher quality. In addition, the up-regulation of the signaling pathways could ensure the quality or precision of homeostatic mechanisms and cell-specific gene expression.

Although they are particularly helpful for analysis, only 19% of all the up-regulated genes (1025 of 5461) were included in currently defined KEGG pathways. We therefore also analyzed other annotation sets. A total of 3222 genes (59.0% of the total number of genes whose expression is up-regulated) could be classified by cellular component (CC) Gene Ontology (GO) terms (table S2). This yielded an impressive top category for “nucleus” with 1014 genes and a *P*_adj_ for the enrichment of 8.3 × 10^−143^, while the nucleus category does not even appear in the list of GO_CC terms for down-regulated genes (table S3). Only 2 of the 187 GO_CC categories for the up-regulated genes appear in the 41 GO_CC categories for the down-regulated genes. Most other terms were consistent with the insights provided by the analysis of KEGG pathways. For example, GO_CC terms associated with structural elements of the mitochondria, including ribosomal proteins, appear exclusively in the up-regulated list, further evidence that PQ stimulates the synthesis of cellular constituents.

### Genes down-regulated by PQ are strongly associated with metabolism, including energy generation

A total of 9.5% of all the down-regulated genes (467 of 4896) were included in defined KEGG pathways. The set of down-regulated genes contrasts markedly with the set of up-regulated genes (Fig. 2 and fig. S3). The top KEGG pathway is metabolic pathways, with 266 genes and a *P*_adj_ of 2.10 × 10^−69^ for the enrichment. Several enriched pathways are linked to amino acid or lipid metabolism, as well as a few that are linked to sugar metabolism. The processes of degradation and breakdown of ingested nutrients also figure prominently, with down-regulation of the lysosome, phagosome, peroxisome, P450-dependent metabolism, and other pathways. All the most important pathways that contribute to energy generation are also down-regulated: carbon metabolism, oxidative phosphorylation, citric acid cycle, fatty acid degradation, pyruvate metabolism, and glycolysis (Fig. 2 and fig. S3). Interrogating the InterPro database of protein domains (ebi.ac.uk/interpro), we also observed a down-regulation of a large family of kinases and phosphatases of unknown function that is known to be expressed in the germ line (*65*) and down-regulated by PQ (*17*).

### Life-span extension by PQ is unaffected by alterations in the AMPK and TOR pathways

Given the observed effects of PQ on metabolic pathways, including energy metabolism, we sought to determine whether the life-span extension by PQ involved metabolic regulators that have previously been shown to be involved in life-span regulation (*1*, *3*, *66*–*68*). Specifically, we asked whether adenosine monophosphate-activated protein kinase (AMPK) function and the target pf rapamycin (TOR) pathway were necessary relays in the life-span extension pathway stimulated by PQ and RDRS. The life span of mutants of *aak-2* (which encodes the regulatory subunit of AMPK) is known to be shortened compared to WT (*66*). We treated the *aak-2* mutants with PQ and observed that PQ was able to increase the life span of *aak-2* mutants to the same extent as that of WT controls, suggesting that PQ does not act upstream of AMPK (fig. S12A). To investigate a possible role of the TOR pathway, we used longevous mutants in genes coding for activities that are involved in the regulation of the TOR pathway. RAGA-1 is a positive regulator of the TOR pathway, and *raga-1(ok386)* mutants are long-lived (*67*). *rsks-1* encodes the ribosomal protein S6 kinase, a target of TOR signaling, and *rsks-1(ok1255)* mutants are long lived (*69*). PQ treatment further increases the life span of both *raga-1* and *rsks-1* mutants (fig. S12, B and C), strongly suggesting that the effect of PQ is independent of the modulation of TOR activity. In both cases, especially *rsks-1*, the effect of PQ appears synergistic to that of the mutation, that is, more than additive. This could indicate that the effects of the TOR pathway and RDRS converge on some downstream process that can only be maximized when both pathways are triggered.

### The pattern of gene expression induced by PQ is an intensification and acceleration of the gene expression changes observed at the end of postembryonic development

**Fig. 3.**
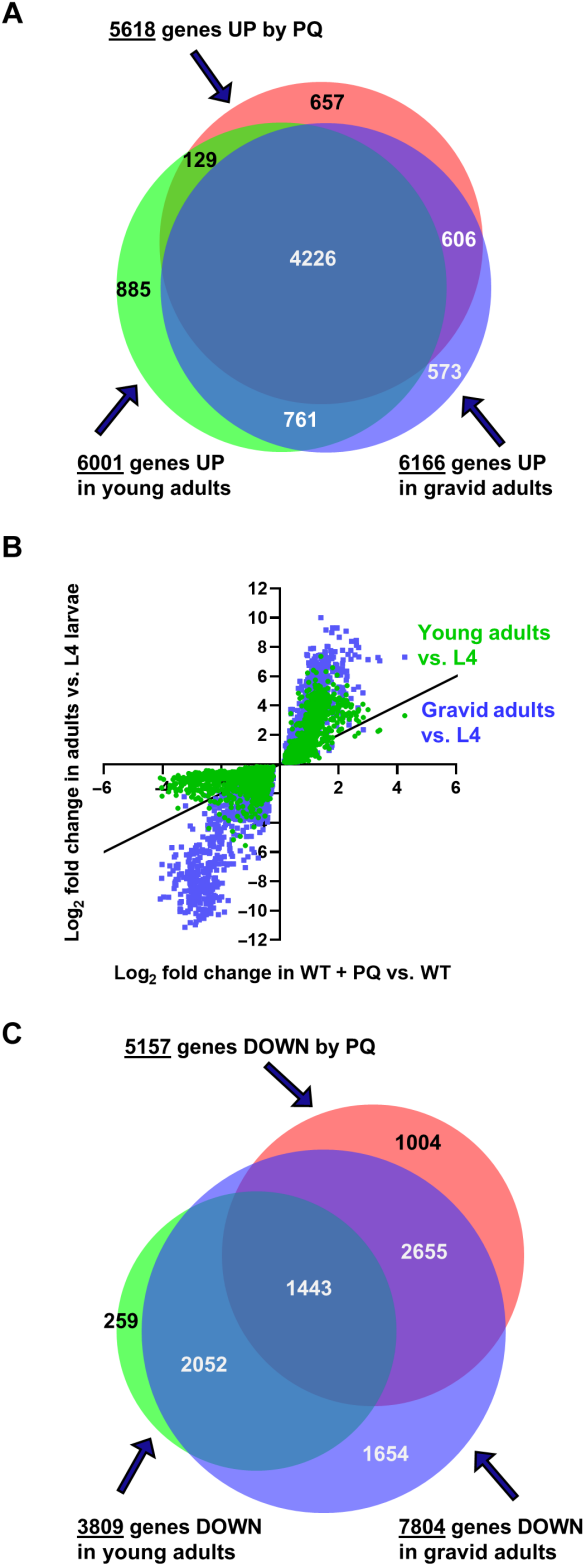
PQ treatment affects a set of genes whose expression changes during development from larvae to adulthood. (**A**) Overlap of genes up-regulated (UP) by PQ treatment compared to untreated young adults, in young adults compared to L4 larvae, and in gravid adults compared to L4 larvae. Log_2_fold changes for the 4226 genes in the triple overlap are plotted in the top right quadrant of (B). (**B**) Correlation plot in which the log_2_ fold changes of the common set of genes changed by three conditions—development from L4 larvae to young adult, from L4 larvae to gravid adults, and by PQ treatment—are plotted. See Materials and Methods. (**C**) Overlap of genes down-regulated (DOWN) by PQ treatment compared to untreated young adults, in young adults compared to L4 larvae, and in gravid adults compared to L4 larvae. Log_2_ fold changes for the 1443 genes in the triple overlap are plotted in the bottom left quadrant of (B). The 55 genes contained in the overlap between genes down-regulated by PQ treatment and in young adults compared to L4 larvae are not shown. The area-proportional Venn diagrams in (A) and (C) are normalized to the area for the 5618 genes up-regulated by PQ in (A).

The large fraction of the genome affected by PQ (>50% of all genes) suggests that it could be modulating a preexisting program of global gene expression. To test this, we scored gene expression in L4 larvae (L4s), in YAs, and in gravid adults (GAs). We then compared the changes we observed to the changes produced by PQ treatment. The Venn diagram in Fig. 3A shows that 6001 genes are up-regulated in YAs compared to L4s, and 6166 are up-regulated in GAs compared to L4s. Both sets are remarkably similar to the set up-regulated by PQ in YAs, with 4226 genes common to all three conditions. We analyzed the degree of up-regulation of the 4226 common genes. The upper right quadrant of the correlation plot shown in Fig. 3B reveals that the common genes are up-regulated up to more than 64-fold from L4s to YAs (green dots, *y* axis). However, the up-regulation of these genes continues further following the YA stage, as shown by the even greater up-regulation from L4s to GAs (blue dots, *y* axis). The effect of PQ on YAs (green dots, *x* axis) is up to a 16-fold up-regulation. PQ therefore intensifies an up-regulation that is already normally occurring between the L4s and adult stages.

Numerous genes are also down-regulated in YAs and GAs compared to L4s (Fig. 3C). Down-regulation happens in two stages: In YAs, 3809 genes are down-regulated compared to L4s. Except for 314 genes, these genes are a subset of the 7804 genes ultimately down-regulated in GAs compared to L4s. In PQ-treated YAs, 1498 genes of the 3809 already down-regulated in YAs compared to L4s are down-regulated further by PQ, and 2655 genes normally down-regulated only at the GA stage are down-regulated prematurely in YAs by PQ. The lower left quadrant of Fig. 3B shows the changes in the degree of down-regulation of the 1443 genes that are down-regulated in all three conditions, with genes down-regulated up to 16-fold by PQ but up to 2000-fold at the GA stage compared to L4s. In summary, PQ treatment intensifies the up-regulation of most of the genes up-regulated from L4 to adults and accelerates as well as intensifies the down-regulation of a large subset (about half) of the genes normally down-regulated by the GA stage compared to L4s.

### Redox regulation through the C118 cysteine of LET-60ras affects the same gene sets as PQ treatment

**Fig. 4.**
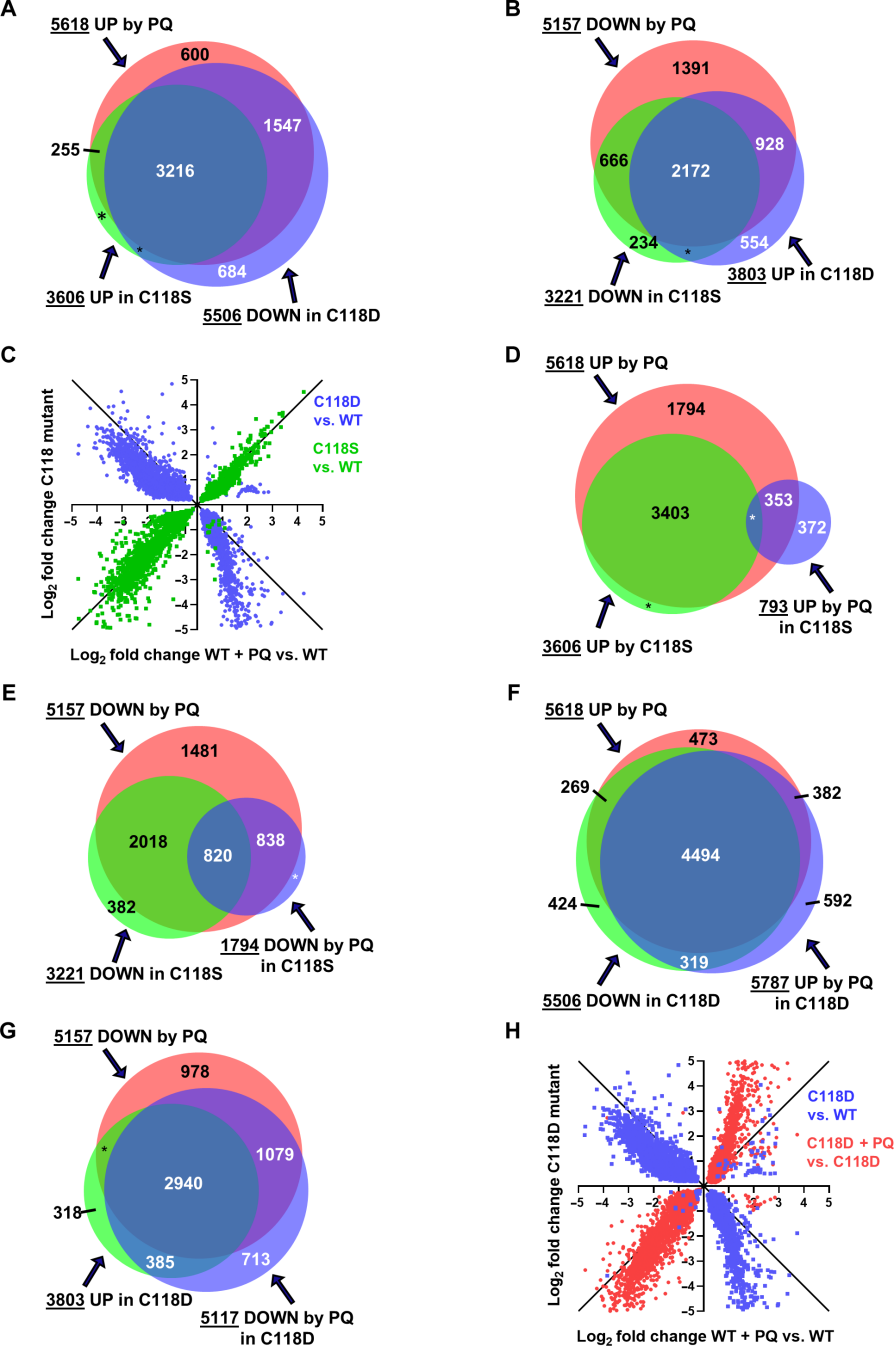
Redox regulation of LET-60ras through C118 affects the same set of genes as treatment of the WT with PQ. (**A**) Overlap of gene sets up-regulated by PQ treatment and in C118S mutants but down-regulated in C118D mutants. (**B**) Overlap of gene sets down-regulated by PQ treatment and in C118S mutants but up-regulated in C118D mutants. (**C**) Correlation plot of the log_2_ fold changes of the set of genes changed in three conditions: In C118S mutants, in C118D mutants, and in WT treated with PQ. See Materials and Methods. (**D**) Overlap of gene sets up-regulated by PQ treatment of the WT and in C118S mutants (compared to WT) and by PQ treatment of C118S mutants (compared to untreated C118S mutants). (**E**) Overlap of gene sets down-regulated by PQ treatment of the WT and in C118S mutants (compared to WT) and by PQ treatment of C118S mutants (compared to untreated C118S mutants). (**F**) Overlap of gene sets up-regulated by PQ treatment of the WT, down-regulated in C118D mutants (compared to WT), and up-regulated by PQ treatment of C118D mutants (compared to untreated C118D mutants). (**G**) Overlap of gene sets down-regulated by PQ treatment of the WT, up-regulated in C118D mutants (compared to WT), and down-regulated by PQ treatment of C118D mutants (compared to untreated C118D mutants). (**H**) Correlation plot of the log_2_ fold changes of the set of genes changed in three conditions: in C118D mutants, in C118D mutants treated with PQ, and in WT treated with PQ. See Materials and Methods. (A, B, and D to G) Area-proportional Venn diagrams normalized to the area for the 5618 genes up-regulated by PQ in (A). An asterisk (*) is used when there are less than 200 genes in the overlap.

The LET-60ras-C118S mutation, which precludes oxidation at that residue, also prevents life-span extension by PQ, and the LET-60ras-C118D mutation, which mimics a partially oxidized cysteine, does not (Fig. 1 and fig. S1). To explore this, we compared the effects of the C118S and C118D mutations on gene expression to those of PQ on the WT (Fig. 4). Given the marked effects that we observed (see below), it is worth reminding the reader here that the LET-60ras mutations that we are studying are not in the gain-of-function *let-60ras* background but in the *let-60ras*(+) background and only alter the one amino acid at residue C118. In C118S mutants, 3606 genes are significantly up-regulated, and, of these, 3471 (96%) are included in the set of 5618 genes up-regulated by PQ (Fig. 4A). Similarly, 3221 genes are down-regulated by C118S, of which 2838 (88%) are included in the set of genes down-regulated by PQ (Fig. 4B). Thus, replacing C118 with a nonoxidizable serine extensively mimics the effect of PQ on gene expression.

The situation is markedly different in C118D mutants. Here, it is the down-regulated gene set that is virtually coextensive with the gene set that is up-regulated by PQ and, conversely, the up-regulated gene set that is mirrored in the gene set down-regulated by PQ. A total of 5506 genes are down-regulated in C118D, and of these, 4763 (87%) are in the set up-regulated by PQ (Fig. 4A). Conversely, 3803 genes are up-regulated, of which 3100 (81.5%) are in the set down-regulated by PQ (Fig. 4B). Of course, many genes are affected in all three conditions (PQ treatment, C118S, and C118D) (Fig. 4, A and B). The degree of change compared to the WT in each condition is shown in a correlation plot for the set of genes that is common to all three conditions (Fig. 4C). The plot shows that the gene expression changes observed in the C118S mutant are very similar to those brought about by PQ treatment in sign and in magnitude. In contrast, in C118D mutants, the gene expression changes are overwhelmingly opposite in sign and different in magnitude (more down-regulated in C118D than they were up-regulated by PQ and less up-regulated than they were down-regulated by PQ).

### PQ treatment affects gene expression markedly in C118D mutants but only minimally in C118S mutants

Although virtually all genes that are up-regulated in C118S mutants are also up-regulated in WT treated with PQ, the converse is not true. A total of 2147 genes that are up-regulated by PQ are not affected by C118S (Fig. 4A), and 2319 genes that are down-regulated by PQ are not down-regulated in C118S mutants (Fig. 4B). Similarly, 855 genes that are up-regulated by PQ are not down-regulated by C118D, and 2057 genes that are down-regulated by PQ are not up-regulated by C118D (Fig. 4B). We therefore tested whether PQ treatment of the mutants would up- or down-regulate those genes that were affected by PQ in WT but were unaffected in the untreated mutants. Figure 4D shows that only 353 of the 2147 (16%) that are unaffected by C118S mutants are up-regulated in response to treatment of C118S with PQ. Furthermore, very few genes (68) up-regulated in C118S are up-regulated to a higher level of expression by PQ treatment of the mutant (shown as an asterisk in Fig. 4D), while 372 genes up-regulated by PQ in the C118S background are not up-regulated by PQ in the WT. In summary, most of the genes that are up-regulated by PQ in the WT are refractory to up-regulation by PQ in the C118S mutant background, whether or not they were up-regulated in untreated C118S compared to WT.

Similar to up-regulated genes, down-regulated genes become refractory to PQ treatment in the C118S background but to a lesser degree (Fig. 4E). A total of 838 of 2319 genes (36%) that are down-regulated by PQ in WT, but are unaffected in untreated C118S mutants, are down-regulated by PQ treatment of the mutants. In addition, 820 of 3221 that are already down-regulated in the mutant are down-regulated further by PQ treatment.

The findings with PQ treatment of C118D are radically different from those with C118S. PQ treatment completely overcomes the reversal produced by the C118D mutation (Fig. 4, F and G). That is, 87% of the genes down-regulated by C118D are now up-regulated following PQ treatment of C118D mutants (4813 of 5506) (Fig. 4F). In addition, 87% of the genes up-regulated in C118D mutants are now down-regulated following PQ treatment of these mutants (3325 of 3803) (Fig. 4G). The effects of PQ on C118D are easily visualized in a correlation plot of the log_2_ fold change for those genes whose expression is changed in all three conditions, that is, in WT treated with PQ, in C118D mutants compared to WT, and in C118D mutants treated with PQ compared to C118D mutants (Fig. 4H). There are 4494 genes up-regulated by PQ in both the WT and C118D backgrounds, and there are 2940 genes that are down-regulated. The correlation plot shows, as we already described above, that gene expression in untreated C118D mutants (red dots) is almost entirely the reverse of that of WT treated with PQ.

In summary, the C118S mutation makes animals refractory to gene expression changes from PQ treatment, but PQ treatment is epistatic to the effects of the C118D mutation. The refractoriness of C118S mutants to PQ treatment is consistent with the failure of PQ treatment to extend their longevity. The ability of PQ to supersede the effect of the C118D mutation is consistent with the life-span extension obtained by treating C118D mutants with PQ (Fig. 1F). In Discussion, we present a model of how gene expression is controlled by RDRS and how we can understand the effects of PQ and of the mutations of C118.

### PQ treatment and LET-60-C118 mutations affect organismal phenotypes, body composition, and energy metabolism

**Fig. 5.**
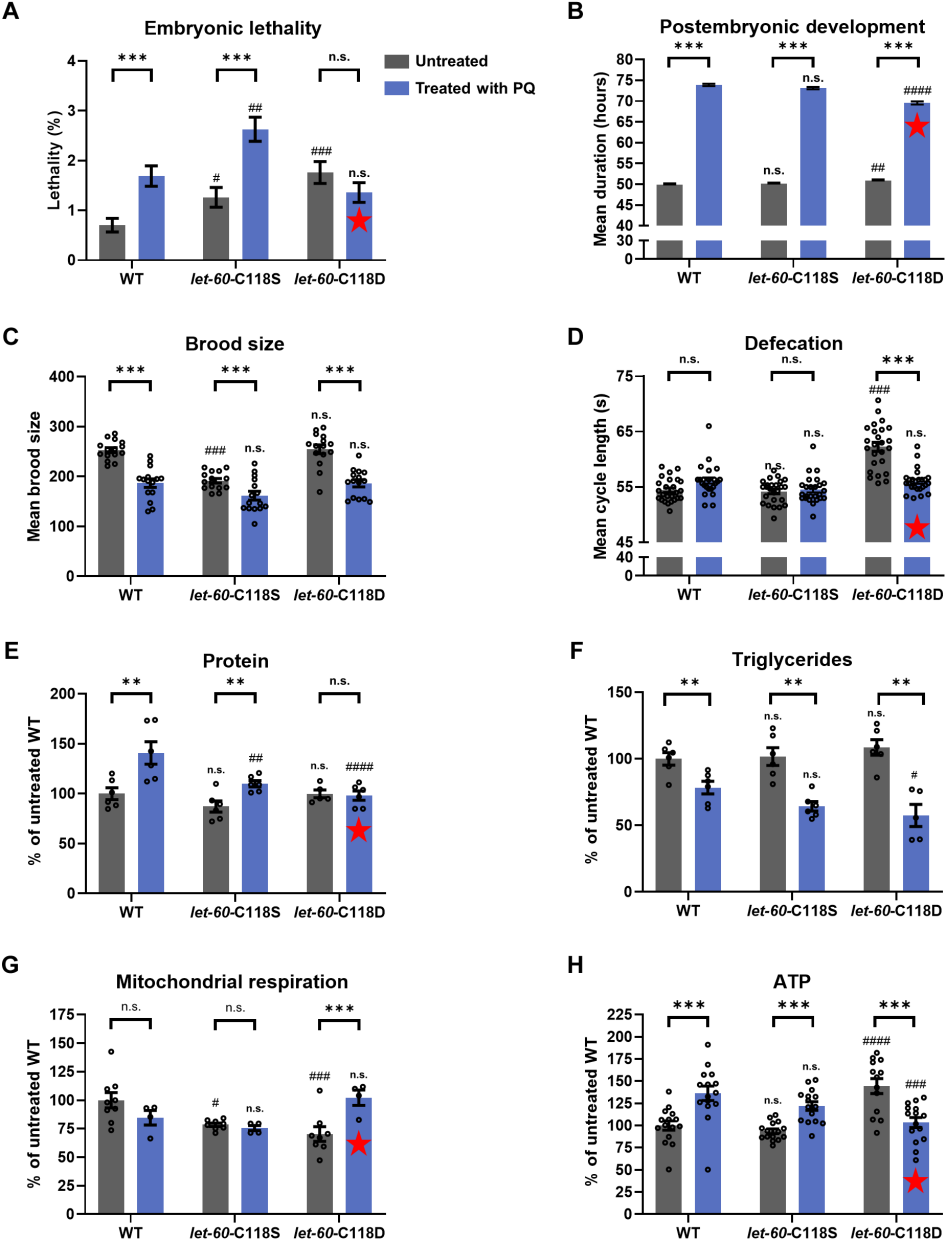
The effect of PQ treatment and LET-60 C118 mutations on organismal phenotypes, body composition, and energy metabolism. (**A**) Percentage of embryonic lethality. Sample sizes for bars from left to right are as follows: 3686, 3904, 3165, 4443, 3573, and 3387. (**B**) Mean duration of development from hatching to adulthood. Sample sizes for bars from left to right are as follows: 86, 115, 106, 107, 73, and 95. (**C**) Mean number of progeny per worm. (**D**) Mean duration of the defecation cycle. (**E**) Protein levels normalized to untreated WT levels. (**F**) Triglyceride levels normalized to untreated WT levels. (**G**) Mitochondrial respiration normalized to untreated WT levels. (**H**) ATP levels normalized to untreated WT levels. Phenotypes of untreated and treated mutants are compared to untreated and treated WT, respectively, and significant differences are indicated above the bars with # (#*P* < 0.05; ##*P* < 0.01; ###*P* < 0.001; ####*P* < 0.0001). For each genotype, the untreated and treated phenotypes were also compared, and significant differences are indicated above the set of bars with asterisks (**P < 0.01; ***P < 0.001). Red stars indicate phenotypes for which treatment of PQ had a different effect on C118D than it did on the other genotypes. For (B) to (H), bars represent means and error bars represent SEM. For (C) to (H), individual points are also plotted; individual points are not plotted for (B), given the large number of points. For (E) to (H), levels are normalized to WT levels, which are given in Materials and Methods.

We first scored organismal phenotypes with and without PQ treatment in WT, C118S, and C118D mutants, including embryonic lethality, growth rate, and fertility, as well as one behavioral rate (defecation) (Fig. 5, A to D). The C118S mutation produced a small increase in embryonic lethality (Fig. 5A) and a decrease in brood size (Fig. 5C) and had no effect on the length of postembryonic development or the defecation rate (Fig. 5, B to D). The C118D mutation also produced a small increase in embryonic lethality and in the length of postembryonic development, as well as a relatively larger decrease in the rate of defecation (Fig. 5, A, B, and D), but had no effect on brood size (Fig. 5C). PQ treatment produced a small increase in embryonic lethality in the WT and in C118S but not in C118D (Fig. 5A), a slowdown of development, and a decrease in brood size in all genotypes (Fig. 5, B and C), but an increase in the defecation rate only of C118D (Fig. 5D). The most salient feature was, consistent with what we found with gene expression (Fig. 4, E and F), that PQ tended to suppress the phenotypic effects produced by C118D (Fig. 5, A and D) or have a lesser effect than on the other genotypes (Fig. 5B). Except for this phenomenon, there is no simple way in which to relate the observed changes in organismal phenotypes to gene expression. This might be because PQ and mutations in C118 only exacerbate, or distort, a complex pattern of gene expression that is part of a developmental program, rather than giving rise to an abnormal novel pattern.

The identities of the genes up-regulated by PQ (Fig. 2 and fig. S3) suggest the possibility of increased protein production. Similarly, the down-regulation of genes involved in lipid metabolism and energy generation might lead to a lower content in lipid reserves. We found that protein and lipid content were unaffected by the mutations but were increased and decreased by PQ, respectively, except in C118D mutants where PQ had no effect on protein levels (Fig. 5, E and F).

For physiological parameters, we observed a minor decrease in mitochondrial respiration in C118S and C118D (Fig. 5G) but an unexpected elevation of adenosine 5′-triphosphate (ATP) levels in C118D (Fig. 5H). PQ had little effect on the WT and C118S respiration but fully suppressed the low respiration in C118D. PQ also elevated ATP levels in WT and C118S but suppressed the elevated levels of C118D (Fig. 5H).

Similar to what we observed for the organismal phenotypes, the C118D mutants displayed the most divergent biochemical parameters, which were suppressed by PQ. Most strikingly, ATP levels were increased in the C118D mutant, whereas respiration was decreased, and these effects were reversed by PQ. How exactly the pattern of gene expression in C118D leads to these phenotypes is unclear.

These findings do not allow us to point to one biochemical parameter that would be particularly crucial for life span. C118S mutants resemble WT for these parameters, but their life span cannot be extended by PQ, while C118D mutants are most different from WT but their longevity remains sensitive to PQ (Fig. 1F). Rather, our findings show that neither more protein, less lipid, more or less ATP, nor more or less respiration is correlated with extended longevity.

Nonetheless, the increased protein and ATP levels triggered by PQ treatment are notable and intriguing. We therefore wondered, as for life span, whether one of the well-studied central metabolic regulators was necessary for these effects. For this, we compared the effect of PQ on these biochemical phenotypes in the WT, *aak-2*, *raga-1*, and *rsks-1* mutants. The increase in protein levels was abolished in all three mutant backgrounds (fig. S12D). Given that PQ-dependent life-span extension is not abolished (fig. S12, A to C), this confirms that the elevated protein levels are not necessary for life-span extension by PQ. Rather, it is consistent with the importance of the TOR pathway in regulating protein synthesis and the importance of the AMPK pathway in monitoring the metabolic state of the cell. The picture is more complex for ATP levels (fig. S12E). Although *aak-2* only has a nonsignificant effect on ATP levels by itself, PQ treatment depresses it further. Similar to protein levels, this confirms that elevated ATP levels are not necessary for life-span extension by PQ. The ATP levels of *raga-1* mutants are like that of the WT, including being elevated by PQ treatment. *rsks-1* mutants are the most divergent, with elevated ATP levels even in the absence of treatment and with no additional effect of PQ. Thus, ATP phenotypes of *raga-1* and *rsks-1* mutants do not, at this time, lead to a clear interpretation of the role of TOR in the effect of PQ on ATP levels.

### RDRS affects gene expression in a way that is opposite to that of a variety of longevity mutants

**Fig. 6.**
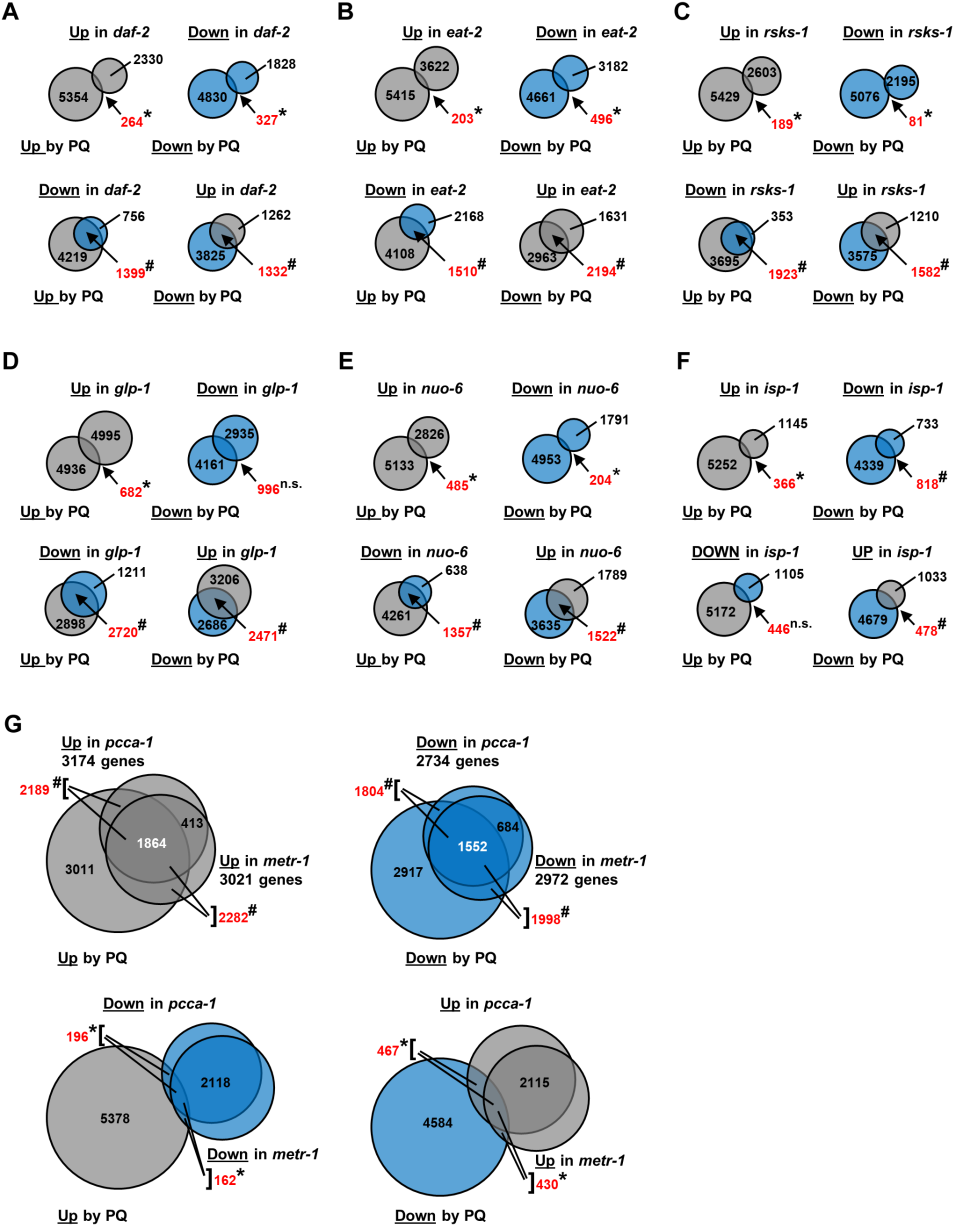
Gene expression changes brought about by RDRS compared to changes observed in longevity mutants. Area-proportional Venn diagrams normalized to the area for the 5618 genes up-regulated by PQ treatment. Gray-filled circles represent up-regulated (Up) genes, and blue-filled circles represent down-regulated (Down) genes. (**A**) Comparison to *daf-2* mutants. (**B**) Comparison to *eat-2* mutants. (**C**) Comparison to *rsks-1* mutants. (**D**) Comparison to *glp-1* mutants. (**E**) Comparison to *nuo-6* mutants. (**F**) Comparison to *isp-1* mutants. (**G**) Comparison to *metr-1* and *pcca-1* mutants. For (A) to (F), the number of genes in the overlap is shown in red. For (G), the number of genes in the overlap with PQ is shown in red, and the number of genes in the triple overlap is shown in white. The bracket indicates the number of genes in the sum of the two overlapping sections to which the lines are pointing. Asterisk (*) indicates that the number of genes in the overlap is significantly less than expected, and # indicates that the number of genes in the overlap is significantly more than expected. Expression data are taken from GSE106672, GSE30505, GSE77109, GSE93724, and GSE43952. *P < 0.001; #*P* < 0.001.

We compared the gene expression changes produced by PQ to those reported for long-lived mutations in *daf-2(e1370)*, *eat-2(ad465)*, *rsks-1(ok1255)*, *glp-1(e2141ts)*, *nuo-6(qm200*), and *isp-1(qm150)* mutants, which alter gene expression by respectively affecting insulin-like signaling, caloric restriction, mTOR signaling, the presence of the germline, and mitochondrial function (*nuo-6* and *isp-1*) (*27*, *28*, *70*, *71*). Figure 6 (A to F) shows that all mutations, except *isp-1*, have effects on gene expression that are, in part, the opposite of those of PQ. Many fewer genes than expected from chance alone are changed in the same direction as they are changed by PQ, and many more genes than expected by chance alone are changed in the opposite direction. In contrast, when gene expression is compared among the long-lived mutants, the genes that are affected in any pair are almost all affected in the same direction (fig. S13). Although it is unique by itself, the PQ pattern thus provides a link between the disparate groups of long-lived mutants by producing gene expression changes that are systematically opposite to that seen in the mutants.

The long-lived *isp-1(qm150)* and *nuo-6(qm200)* mutants carry point mutations in subunits of the mitochondrial respiratory chain and have much in common (*6*, *17*). However, their pattern of gene expression is quite dissimilar (*27*), and that of *isp-1* does not show the reversed pattern discussed above. Understanding differences and similarities between the biology of *isp-1* and *nuo-6* mutants might therefore be key to understanding the unexpected pattern of gene expression that we describe.

Interrogating the Gene Expression Omnibus (GEO) database (ncbi.nlm.nih.gov/geo/), we compared the RDRS pattern of gene expression changes for available datasets for *C. elegans*. We found that the effects of PQ on gene expression most closely resemble those of *metr-1* and *pcca-1* mutants fed on the bacteria *Comamonas* DA1877 (Fig. 6G and fig. S15) (*72*). *metr-1* and *pcca-1* affect methionine metabolism and branched chain amino acid (BCAA) breakdown, respectively, and have an abnormal transcriptional response when fed *Comamonas*. This suggests that specific alterations in amino acid metabolism can affect part of the same developmental gene expression program as PQ. BCAA catabolism has previously been linked to longevity in worms (*73*).

## DISCUSSION

### RDRS plays a crucial role in modulating global gene expression

**Fig. 7.**
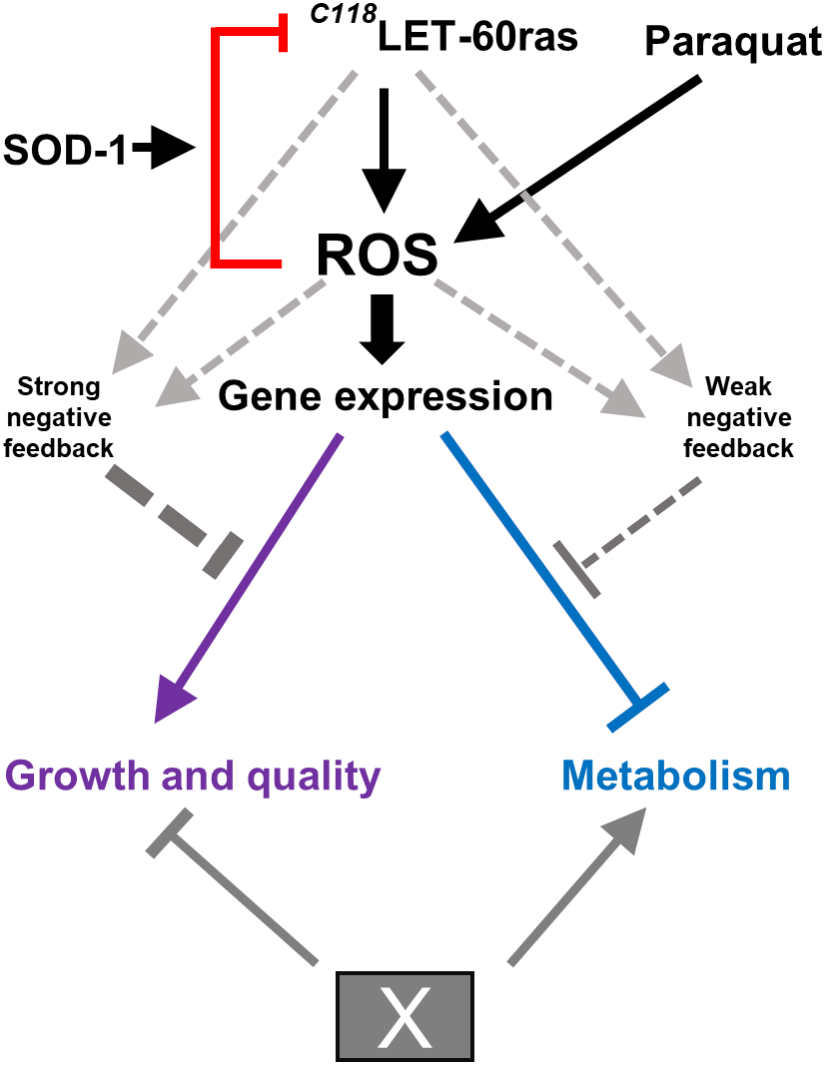
A model of how RDRS affects gene expression. ROS generated by PQ acts downstream of LET-60ras to enhance RAS-dependent ROS signaling (RDRS). RDRS up-regulates genes associated with growth and the high-quality synthesis of cellular constituents and down-regulates genes necessary for degradation, intermediary metabolism, and energy generation. ROS also directly regulates RAS by oxidation of cysteine C118 of LET-60ras. This effect, which requires SOD-1, appears to act as negative feedback from RDRS (red arrow). The gray dashed arrows represent negative transcriptional feedback that has been observed to affect RAS signaling in other systems and whose action on RDRS in *C. elegans* is also evident in our observations. X represents an unknown pathway that also affects global gene expression but with effects opposite to those of RDRS. See Discussion and fig. S14 for further description of the model.

Our findings reveal that mitochondrial superoxide generation regulates global gene expression and life span by modulating RDRS. Figure 7 presents a schematic model, and figure S14 and its legend elaborate on how the model is derived from the experimental results. RAS signaling is well known to stimulate the generation of ROS that serve as a signaling relay. Our model is based on the central observations that PQ treatment affects the same sets of genes as *let-60ras*-C118 mutants (Fig. 4, A to C), but that PQ does not act on life span via the redox state of C118 (Fig. 1, E and F). C118S mutants are not short-lived but refractory to life-span extension by PQ, and C118D mutants are not long-lived and remain sensitive to life-span extension by PQ. This led us to formulate the hypothesis that ROS generated by PQ enhance a RAS-dependent downstream ROS signal that affects global gene expression. RDRS up-regulates genes associated with growth and the high-quality synthesis of cellular constituents and down-regulates genes necessary for intermediary metabolism and energy generation.

Our previous findings on the effects of C118 oxidation on vulval development indicated that C118 oxidation negatively regulates LET-60ras activity (*47*). Because C118 oxidation inhibits LET-60ras signaling, we speculate that it represents negative feedback from downstream RAS-dependent ROS. Removal of a negative feedback is expected to result in stimulation of RAS signaling, as we have also observed for vulval development, and which explains why the pattern of gene expression produced by C118S resembles that produced by PQ.

The effect of the C118D mutation can be analyzed similarly. Replacement of C118 with aspartic acid (D) leads to strong inhibition of LET-60ras activity (*47*). This results in a pattern of gene expression changes that is the reverse of that of C118S and, even more so, the reverse of that of PQ treatment of the WT. How does lower-than-normal LET-60ras activity in C118D result in a pattern of gene expression that is reversed compared to the effect of higher-than-normal LET-60ras activity? We postulate the existence of another major pathway that affects global gene expression but acts in the opposite direction from RAS (the pathway is designated X in Fig. 7). The nature of the pathway is currently unknown, but an antagonistic pathway with different inputs to balance gene expression is not unexpected. When C118D mutants are treated with PQ, this bypasses the weakened LET-60ras signaling with an intense downstream ROS signal that can overcome the effect of X and produces a pattern similar to that produced by PQ treatment of the WT.

Negative transcriptional feedbacks are well documented for many signaling pathways, including for RAS and its effectors (*74*, *75*). These act to maintain signaling intensity within a particular range. Such negative feedback loops (indicated with gray dashed arrows in Fig. 7) are consistent with the observation that C118S mutants are strongly refractory to the action of PQ on gene expression and life span. Our interpretation is that the absence of the normal feedback provided by oxidation of C118 leads to the chronic activation of LET-60ras and chronic enhanced ROS generation. This activates a signal-dampening mechanism that prevents the artificial elevation of ROS by PQ to have major effects on gene expression in C118S mutants. The dampening of the effect of PQ in the mutants appears to be more pronounced for up-regulated genes than for down-regulated genes (Fig. 4, D and E).

### How do changes in gene expression lead to longevity?

We have shown how modulating RDRS affects the expression of half of the genome, yet a clear pattern still emerges (Fig. 2 and fig. S3). Many of the genes and well-understood processes that are up-regulated by PQ are involved in expressing genes correctly and ensuring that the resulting molecules are error free. To designate this pattern, we use the moniker “growth and quality.” Quality has been considered an explanation for the longer life spans of some species with lower metabolic rates. For example, longer-lived species have better DNA repair mechanisms (*76*). Whether decreasing errors in gene expression or better turnover of damaged proteins could lead to increased life span is also being explored experimentally by different means (*77*, *78*).

The genes and processes that are down-regulated are involved in energy generation from the breakdown of sugars, lipids, and amino acids from ingested nutrients, which we call “metabolism.” This low metabolism leads to lower fat reserves. Thus, RDRS controls the balance between the two major things that organisms have to do: develop and grow, and extract resources from the environment to produce offspring, for which metabolism is needed. Of course, RDRS does not control by itself all the underlying processes, but it appears to play a crucial role in the balance between “quality” and metabolism.

Low metabolism is associated with long life spans in larger species. Whether this could be causal is still debated. Several hypotheses are possible as to how the WT level of metabolism limits life span. One possibility is that metabolism serves to produce storage molecules such as fat to help with progeny production in an unpredictable environment. This is available energy that is not used for quality. Another possibility is that metabolism produces side effects, such as toxic chemicals, that limit survival, such as ROS in the wrong place and the wrong time, or toxic intracellular lipids.

We have shown that PQ intensifies a program of change in gene expression that is at work during the transition from larvae to fertile adult (Fig. 3). Thus, part of the normal pattern might correspond to ensuring that quality materials are stored in the eggs. We speculate that PQ treatment might be extending part of this program to the soma, thus leading to extended longevity.

One limitation of our study is that we cannot determine which subset of changes in expression among the large number of changes produced by PQ is necessary for longevity. However, it is clear that a partial pattern, as observed in C118S mutants, whether treated with PQ or not, is not sufficient. Unfortunately, the observations with C118D do not resolve the issue, because treatment of this mutant with PQ, which increases the longevity of the mutant, produces mostly the same pattern of changes as treatment of the WT. Nonetheless, it seems unlikely that a small subset of genes is responsible for the effects on life span rather than the major shifts that affect growth and quality and metabolism.

### Could changes in gene expression that favor longevity in worms favor tumorigenesis in vertebrates?

We describe a pathway that starts with increased mtROS generation and has widespread effects on gene expression. mtROS have been shown to be required for Kras-induced cell proliferation and tumorigenesis (*44*). In general, the role that ROS play in tumor cells is hotly debated but not resolved.

## MATERIALS AND METHODS

### Experimental design

The objective of our study was to identify the mechanism(s) by which elevating mitochondrial superoxide generation increases life span, which is a previously documented phenomenon. For this, we sought to compare the gene expression changes and phenotypic effects produced by treatment with the prooxidant PQ, a compound known to boost superoxide generation in the mitochondrial matrix. Our previous work suggested that PQ can act on the C118 cysteine of the *C. elegans* Kras homolog LET-60ras. C118 is also known to be redox sensitive in vertebrates. We explored how PQ affects ROS levels in the cytoplasm and how ROS, in turn, interact with RAS, both upstream, via oxidation of C118, and downstream, via intensification of RAS-dependent ROS generation. We then studied gene expression as the output of the system that will ultimately lead to changes in life span. The gene expression datasets also allowed us to compare the effect of drug treatments, genetic interventions on *let-60ras*, and previously published genetic interventions on other genes.

### General worm maintenance

All strains were maintained at 20°C on solid nematode growth medium (NGM) and were fed *Escherichia coli* OP50. The Bristol strain N2 was used as the WT. The following mutant strains were used: LGI: *nuo-6(qm200)* and *sod-2(ok1030)*; LGII: *sod-1(tm783)*, *sod-5(tm1146)*, *ctl-1(ok1242)*, *ctl-2(ok1137)*, *ctl-3(ok2042)*, and *raga-1(ok386)*; LGIII: *sod-4(gk101)* and *rsks-1(ok1255)*; LGIV: *let-60(qm226)* (referred to as *let-60*-C118S in the paper) and *let-60(qm228)* (referred to as *let-60-*C118D in the paper); and LGX: *sod-3(tm760)* and *aak-2(ok524).* Double-mutant strains were generated using standard genetic methods and were verified using polymerase chain reaction and restriction enzyme digest where applicable.

PQ (also called methyl viologen dichloride hydrate; Sigma-Aldrich, 856177) was dissolved in water and stored at 4°C. It was added to the NGM just before pouring the plates to a final concentration of 0.1 mM. Because bacteria do not grow as quickly on PQ plates, instead of seeding them directly onto the plates, OP50 bacteria grown on regular NGM plates were transferred onto NGM-PQ plates using a platinum loop. Control NGM plates for the experiment were treated in the same fashion. In some experiments, 5-fluorodeoxyuridine (FUdR; Sigma-Aldrich, F0503) was dissolved in water and stored at −20°C. It was added to the NGM just before pouring the plates to a final concentration of 50 μM.

### Aging

Gravid adult worms were transferred to NGM for a 3-hour limited egg-laying. Once the hatched worms reached the young adult stage and had completed vulval development, they were transferred onto experimental plates. The worms were transferred every other day during the egg-laying period and then once or twice a week. Worms were monitored every other day until dead. Worms showing phenotypes of internal hatching, gut extrusion, or protruding vulva were not included in the study and were replaced from a backup pool. For aging experiments presented in Fig. 1 (A to C), where adult life span is reported, the first day of adulthood was considered day 0. For PQ experiments, the limited laying was performed on plates containing 0.1 mM PQ, and the progeny was transferred to plates containing 0.1 mM PQ once they reached adulthood. These experiments measured adult life span to avoid confounding effects from PQ on developmental rate. For aging experiments presented in Fig. 1 (E and F), day 0 was the day of the limited laying. Once the hatched worms reached the young adult stage and had completed vulval development, they were transferred onto the experimental plates, which contained 50 μM FUdR and 0.1 mM PQ where indicated. Worms were transferred onto freshly prepared FUdR plates once a week during the experiment. Samples sizes and statistics for all aging experiments are presented in table S1.

### RNA sequencing

Worms were collected at the young adult stage before they began laying eggs, unless otherwise indicated. RNA was extracted using QIAzol Lysis Reagent and RNeasy Spin columns (QIAGEN). Sequencing libraries were prepared using the KAPA Stranded or RNA HyperPrep kits for the Illumina platform. Libraries were sequenced using the Illumina NextSeq500 with a coverage of ~28 million fragments per sample (~56 million paired-end reads). Sequences were trimmed for sequencing adapters and low-quality 3′ bases using Trimmomatic version 0.35 (*79*) and aligned to the reference *C. elegans* genome version WBcel235 (gene annotation from Ensembl version 88) using STAR version 2.5.1b (*80*). Gene expression was obtained both as read counts directly from STAR as well as computed using RSEM (*81*) to obtain gene and transcript level expression, in either transcripts per million (TPM) or fragments per kilobase of transcript per million fragments mapped (FPKM) values. DESeq2 version 1.22.2 (*82*) was then used to normalize gene read counts. We used a significance level cutoff of *P* < 0.05, using *P* values adjusted by the Benjamini and Hochberg method (*83*). RNA-seq data have been submitted to GEO (GSE200912).

### Developmental and behavioral phenotypes

#### Embryonic lethality

Eggs produced by an overnight limited laying on NGM-control or NGM-PQ plates were transferred onto fresh NGM-control or NGM-PQ plates. Worms that had not hatched 24 to 48 hours later were scored as dead embryos.

#### Postembryonic development

Eggs produced by an overnight limited laying on NGM-control or NGM-PQ plates were transferred onto fresh NGM-control or NGM-PQ plates for a 3-hour “limited hatching.” At the end of the interval, worms were considered to be 1.5 hours old. Worms were monitored every hour until they reached adulthood.

#### Brood size

Worms were transferred to NGM-control or NGM-PQ plates for an overnight limited laying. When the progeny reached the L4 stage, they were placed singly on NGM-control or NGM-PQ plates. The number of viable progeny produced by single worms was counted.

#### Defecation

Worms were transferred to NGM-control or NGM-PQ plates for an overnight limited laying. When the progeny reached the young adult stage, they were scored for three consecutive defecation cycles at 20°C. The average of the three cycle lengths was considered as the defecation cycle length for that animal.

### Quantification of protein levels

A total of 200 young adult worms were picked into individual 1.5-ml Eppendorf tubes and washed with M9 buffer before being frozen in 100 μl of ultrapure water at −80°C overnight. The next day, worms were thawed, and 4× lysis buffer [250 mM tris-HCl (pH 6.8), 40% glycerol, and 8% SDS] was added. The worms were subjected to three cycles of freeze-thaw, followed by two cycles of sonication (Qsonica XL-2000 sonicator at the power setting of 8 for 10 s followed by 20 s on ice) to create a lysate. The samples were centrifuged at maximum speed, and the supernatant was collected. Five microliters of the supernatant was used to determine protein levels using the detergent compatible (DC) protein assay (Bio-Rad). Protein levels in Fig. 5E were normalized to the untreated WT level of 145.38 μg.

### Quantification of triglyceride levels

Young adult worms were collected by washing them off two 6-cm plates. Worms were washed with M9 buffer before being resuspended in 100 μl of M9 buffer containing 0.05% Tween 20 solution in 2-ml sample tubes (QIAGEN). Worms were homogenized for 5 min at 50 Hz in a TissueLyser LT (QIAGEN). Samples were centrifuged at maximum speed, and the supernatant was collected. For each sample, measurements were taken using 3 and 6 μl of the supernatant mixed with 300 μl of Infinity Triglycerides Reagent (Thermo Fisher Scientific). Samples were transferred to a 96-well plate to determine triglyceride levels by measuring the optical density at the primary wavelength of 500 nm and secondary (reference) wavelength of 660 nm. Triglyceride levels were normalized to protein levels. Protein levels were determined using 5 μl of the supernatant in a bicinchoninic acid (BCA) protein assay (Bio-Rad). Triglyceride levels in Fig. 5F were normalized to the untreated WT level of 17.16 μg/mg protein.

### Quantification of ATP levels

Young adult worms were collected by washing them off two 6-cm plates. Worms were washed with M9 buffer before being frozen in 100 μl of ultrapure water at −80°C overnight. The next day, frozen worms were immersed in boiling water for 20 min to release ATP and destroy ATPase activity. The samples were placed on ice for 5 min before being centrifuged at maximum speed at 4°C. The supernatant was collected, and 5 or 10 μl of the supernatant was used to determine ATP levels using the ATP Bioluminescence Assay Kit CLS II (Sigma-Aldrich). Samples were loaded into a 96-well white opaque polystyrene plate (Pierce), and luminescence was measured in a plate reader (TECAN, Infinite M1000). ATP levels were normalized to protein levels. Protein levels were determined using 12.5 μl of the supernatant in a BCA protein assay (Bio-Rad). ATP levels in Fig. 5H were normalized to the untreated WT level measured on the same day, which averaged 26.46 pmol/μg protein.

### Mitochondrial respiration

Young adult worms were collected by washing them off plates. Worms were washed with M9 buffer and loaded into the 2-ml chamber of the Oroboros Oxygraph 2K. The number of worms in 10 aliquots of 25 μl of the worm suspension was counted to determine the total number of worms in the chamber. Oxygen consumption was measured for at least 10 min at 20°C, followed by injection of sodium azide (final concentration of 10 mM). Residual oxygen consumption after addition of sodium azide was subtracted from the total oxygen consumption to determine the rate of mitochondrial respiration. Mitochondrial respiration rate was normalized to the worm number in the chamber, which averaged around 1000 worms. Mitochondrial respiration rates in Fig. 5G were normalized to the untreated WT rate measured on the same day, which averaged 16.89 pmol/s*μl per worm.

### Correlation plots

We have used correlation plots in Figs. 3B and 4 (C and H) to compare the log_2_ fold changes of a common set of genes changed in three conditions. In Fig. 3B, for each gene, the log_2_ fold change from L4 larvae to YA versus the log_2_ fold change induced by PQ (compared to untreated WT) is plotted in green; the log_2_ fold change from L4 larvae to GA versus the log_2_ fold change induced by PQ (compared to untreated WT) is plotted in blue. In Fig. 4C, for each gene, the log_2_ fold change induced by C118S (compared to WT) versus the log_2_ fold change induced by PQ (compared to untreated WT) is plotted in green; the log_2_ fold change induced by C118D (compared to WT) versus the log_2_ fold change induced by PQ (compared to untreated WT) is plotted in blue. In Fig. 4H, for each gene, the log_2_fold change induced by C118D (compared to WT) versus the log_2_fold change induced by PQ (compared to untreated WT) is plotted in blue; the log_2_fold change induced by PQ on C118D (compared to untreated C118D) versus the log_2_fold change induced by PQ (compared to untreated WT) is plotted in pink.

### Bioinformatics

Overlaps in gene expression were identified using BioVenn (*84*). To compare gene expression changes to published data, lists of significantly changed genes (data file S1) were obtained from the National Center for Biotechnology Information (NCBI) Geo using GEO2R (www.ncbi.nlm.nih.gov/geo/geo2r) and a significance level cutoff of *P* < 0.05 using *P* values adjusted by the Benjamini and Hochberg (false discovery rate) method. Gene names were converted to WormBase IDs using the SimpleMine tool in WormBase (https://wormbase.org/tools/mine/simplemine.cgi). Gene names that could not be converted were excluded from the analysis.

### Statistics

For aging experiments (Fig. 1), means were compared using one-way analysis of variance (ANOVA) and Sidak’s multiple comparison test. Phenotypes in Fig. 5 (B to H) were compared to the control using one-way ANOVA and Dunnett’s multiple comparison test, and the effects of PQ on each genotype were compared using multiple *t* tests and the Bonferroni correction for multiple comparisons. Fisher’s exact test was used for comparing embryonic lethality (Fig. 5A) and overlaps in Venn diagrams (Fig. 6). The expected number of gene overlaps for Fig. 6 was calculated using the following equation: (*G*1 * *G*2)/*N*, where *G*1 is the number of genes in the first group, *G*2 is the number of genes in the second group, and *N* is the total number of genes. We estimated *N* to be 18,000 for data from gene arrays and 20,000 for data from RNA-seq. Statistics were calculated using either GraphPad Prism 9 (version 9.3.1) or GraphPad QuickCalcs (graphpad.com/quickcalcs/contingency2/), which was used for Fisher’s exact test.

## Notes

View/request a protocol for this paper from *Bio-protocol*.
