# Supplementary material for: Stimulation of RAS-dependent ROS signaling extends longevity by modulating a developmental program of global gene expression
Source: Sci Adv. 2022 Nov 30;8(48):eadc9851. doi: 10.1126/sciadv.adc9851 (PMC9710873; doi:10.1126/sciadv.adc9851)
Supplement: Supplementary file 1 — Figs. S1 to S14 Tables S1 to S3 [file sciadv.adc9851_sm.pdf]

Science Advances
AAAS

Supplementary Materials for

Stimulation of RAS-dependent ROS signaling extends longevity by modulating a developmental program of global gene expression

Robyn Branicky *et al.*

Corresponding author: Siegfried Hekimi, siegfried.hekimi@mcgill.ca

Sci. Adv. **8**, eadc9851 (2022)
DOI: 10.1126/sciadv.adc9851

The PDF file includes:

Figs. S1 to S14
Tables S1 to S3
Legend for data file S1

Other Supplementary Material for this manuscript includes the following:

Data file S1

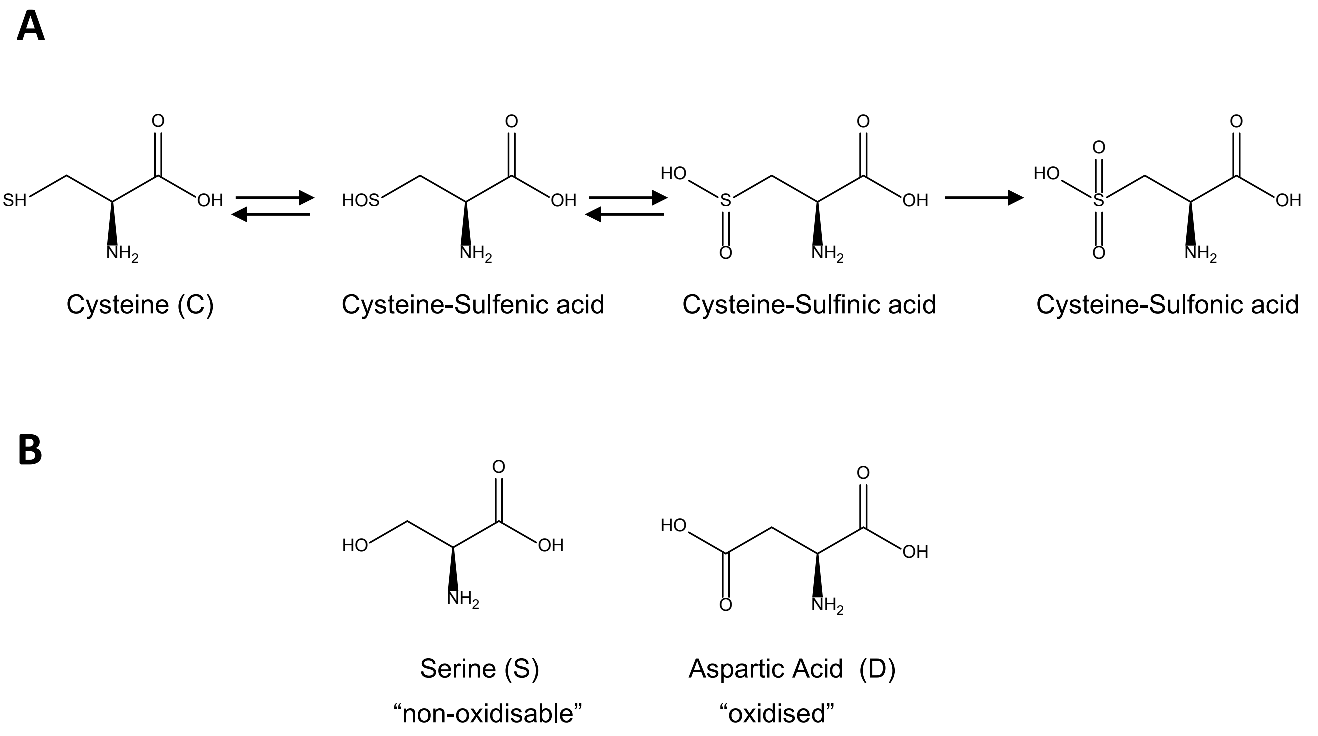

Fig. S1. Oxidative modifications of cysteine

(**A**) The initial reaction of cysteine (C) with oxidants yields sulfenic acid (SOH). SOH can be further oxidized to generate sulfinic (SO_2H) and sulfonic (SO_3H) acid. (**B**) Serine (S) is similar to cysteine but cannot be oxidized, whereas aspartic acid (D) mimics sulfinic acid, a partially oxidized form of cysteine.

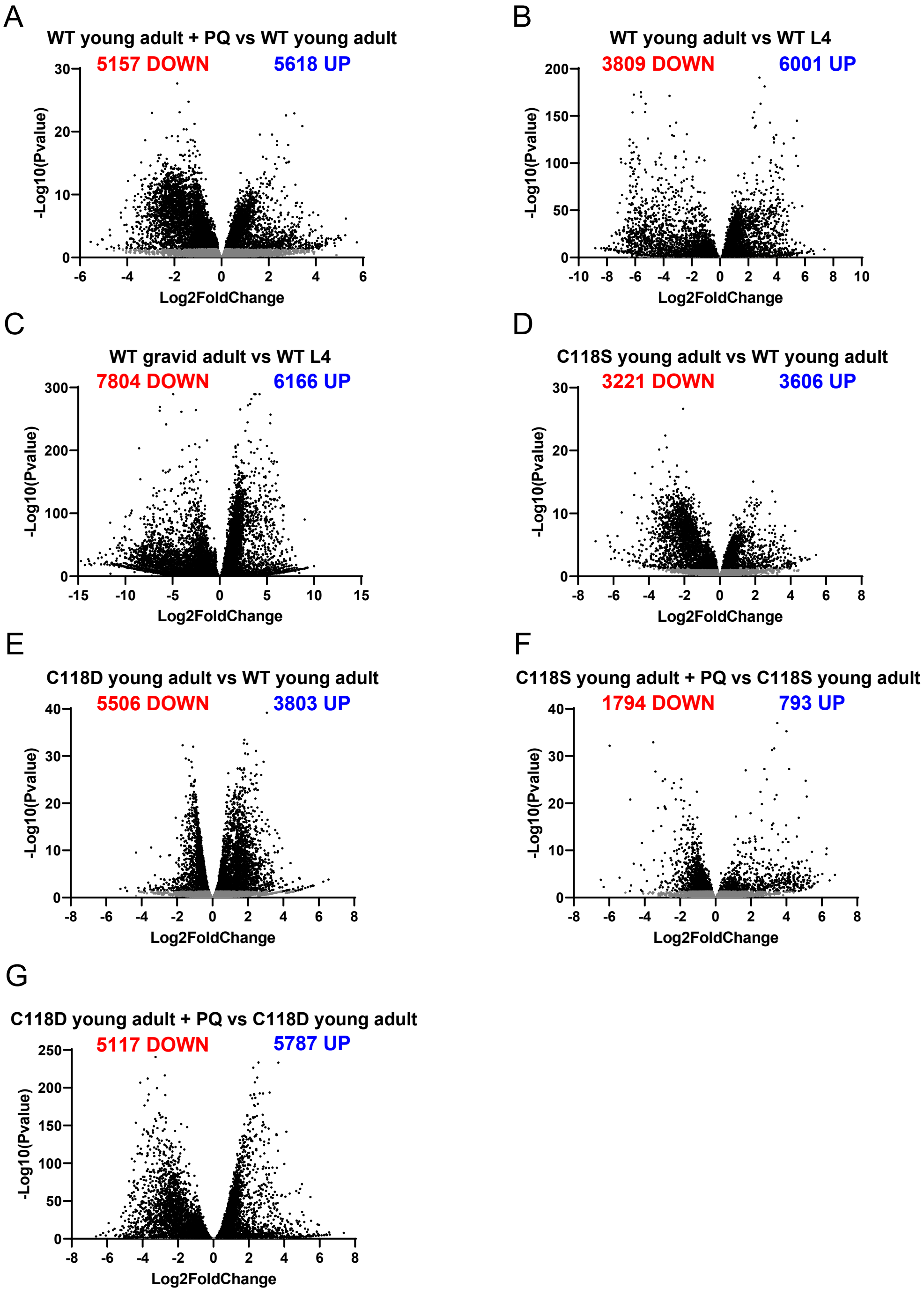

Fig. S2. Volcano Plots of RNAseq data

Log2fold changes in gene expression are plotted against -Log10(P value) where the P values are adjusted for multiple comparisons (Padj) using the method of (*83*). Points in black are significantly different between the conditions indicated in the graph titles (at a confidence level of Padj <0.05). Points in grey are not significantly different. In panels (**B**), (**C**) and (**G**), the grey points are not visible because of the data range plotted. Axes have been chosen to best represent the data. The number of genes that are significantly down-regulated and up-regulated are given in red and blue, respectively.

Poly-saccharide biosynthesis	Signalling	Machinery of gene expression	Quality Control of Cellular Constituents	DNA synthesis and repair	Other	Amino Acid Metabolism	Lipid Metabolism	Sugar Metabolism	Energy Generation	KEGG Pathway	Count	P-Value	Benjamini
										**Genes upregulated in WT vs WT+PQ [includes 19% of upregulated genes]**			
										Spliceosome	101	6.20E-41	7.30E-39
										RNA transport	94	3.00E-30	1.80E-28
										mRNA surveillance pathway	55	1.10E-17	4.30E-16
										Endocytosis	75	1.90E-16	5.50E-15
										Ubiquitin mediated proteolysis	58	3.60E-13	8.50E-12
										Proteasome	35	1.10E-12	2.20E-11
										RNA degradation	38	6.60E-12	1.10E-10
										Nucleotide excision repair	31	1.00E-11	1.50E-10
										Pyrimidine metabolism	50	1.50E-11	1.90E-10
										Basal transcription factors	30	1.60E-10	1.80E-09
										DNA replication	29	4.10E-10	4.40E-09
										RNA polymerase	23	5.00E-09	4.90E-08
										Fanconi anemia pathway	22	1.40E-08	1.30E-07
										Protein processing in endoplasmic reticulum	71	1.20E-07	1.00E-06
										Ribosome biogenesis in eukaryotes	49	3.90E-07	3.10E-06
										Mismatch repair	16	5.30E-06	3.90E-05
										Base excision repair	13	2.00E-05	1.40E-04
										Homologous recombination	14	3.70E-05	2.40E-04
										FoxO signaling pathway	30	1.30E-04	8.00E-04
										Notch signaling pathway	14	1.30E-04	8.00E-04
										TGF-beta signaling pathway	22	7.60E-04	4.30E-03
										Glycosylphosphatidylinositol(GPI)-anchor biosynthesis	10	1.50E-03	8.30E-03
										Glycosaminoglycan biosynthesis - heparan sulfate / heparin	8	9.10E-03	4.50E-02
										mTOR signaling pathway	17	9.40E-03	4.50E-02
										Dorso-ventral axis formation	11	9.50E-03	4.50E-02
										Amino sugar and nucleotide sugar metabolism	19	1.10E-02	5.10E-02
										Wnt signaling pathway	29	1.20E-02	5.10E-02
										Purine metabolism	47	1.70E-02	6.70E-02
										ErbB signaling pathway	19	1.70E-02	6.70E-02
										Regulation of autophagy	11	1.80E-02	7.00E-02
										Glycosaminoglycan biosynthesis - chondr. sulfate/dermatan sulfat	6	1.90E-02	7.30E-02
										Jak-STAT signaling pathway	8	2.10E-02	7.90E-02
										Non-homologous end-joining	5	4.80E-02	1.70E-01
										SNARE interactions in vesicular transport	13	5.30E-02	1.90E-01
										Inositol phosphate metabolism	14	6.50E-02	2.20E-01
										N-Glycan biosynthesis	16	6.60E-02	2.20E-01
										Insulin resistance	17	7.60E-02	2.40E-01
										**Genes downregulated in WT vs WT+PQ [includes 9.5% of downregulated genes]**			
										Metabolic pathways	266	1.90E-71	2.10E-69
										Biosynthesis of antibiotics	82	3.20E-24	1.70E-22
										Carbon metabolism	58	1.50E-22	5.60E-21
										Oxidative phosphorylation	61	4.50E-22	1.20E-20
					Degradation					Lysosome	48	4.20E-20	9.20E-19
										Valine, leucine and isoleucine degradation	28	1.00E-13	1.90E-12
										Biosynthesis of amino acids	37	2.00E-13	3.10E-12
										Glyoxylate and dicarboxylate metabolism	22	5.80E-11	7.90E-10
										Propanoate metabolism	17	2.50E-09	3.00E-08
					Degradation					Peroxisome	30	5.70E-09	6.10E-08
										Cysteine and methionine metabolism	20	6.20E-09	6.10E-08
										Tryptophan metabolism	19	1.20E-08	1.10E-07
										Fatty acid metabolism	24	3.30E-08	2.70E-07
					Degradation					Phagosome	26	1.40E-07	1.10E-06
										Calcium signaling pathway	20	1.80E-07	1.30E-06
										beta-Alanine metabolism	14	2.70E-07	1.80E-06
										Citrate cycle (TCA cycle)	18	1.30E-06	8.10E-06
										Fatty acid degradation	21	1.90E-06	1.10E-05
										Glycine, serine and threonine metabolism	15	5.80E-06	3.20E-05
					Degradation					Drug metabolism - cytochrome P450	18	5.90E-06	3.20E-05
										Alanine, aspartate and glutamate metabolism	16	8.80E-06	4.60E-05
										Pyruvate metabolism	14	1.20E-05	5.50E-05
										Arginine and proline metabolism	14	1.20E-05	5.50E-05
										Butanoate metabolism	12	4.70E-05	2.10E-04
										2-Oxocarboxylic acid metabolism	11	4.80E-05	2.10E-04
										Fructose and mannose metabolism	11	9.40E-05	3.80E-04
										Pentose and glucuronate interconversions	11	9.40E-05	3.80E-04
										Glutathione metabolism	17	1.40E-04	5.30E-04
										Glycolysis / Gluconeogenesis	16	3.70E-04	1.40E-03
										Biosynthesis of unsaturated fatty acids	9	3.80E-04	1.40E-03
										Cyanoamino acid metabolism	6	4.50E-04	1.60E-03
					Degradation					Metabolism of xenobiotics by cytochrome P450	14	4.90E-04	1.70E-03
										Sphingolipid metabolism	13	6.60E-04	2.20E-03
										Lysine degradation	14	6.90E-04	2.20E-03
										Ascorbate and aldarate metabolism	9	7.10E-04	2.20E-03
										ABC transporters	9	1.20E-03	3.60E-03
										Fatty acid elongation	9	1.20E-03	3.60E-03
										Taurine and hypotaurine metabolism	5	2.50E-03	7.00E-03
										Arginine biosynthesis	8	2.50E-03	7.10E-03
										MAPK signaling pathway	18	6.00E-03	1.60E-02
										Other glycan degradation	8	6.40E-03	1.70E-02
										Inositol phosphate metabolism	10	1.20E-02	3.20E-02
										Retinol metabolism	8	1.30E-02	3.40E-02
					Degradation					Drug metabolism - other enzymes	10	1.60E-02	4.00E-02
										Phosphatidylinositol signaling system	11	1.80E-02	4.40E-02
										Wnt signaling pathway	16	2.20E-02	5.30E-02
										One carbon pool by folate	6	2.60E-02	5.90E-02
										Sulfur metabolism	6	2.60E-02	5.90E-02
										Nitrogen metabolism	6	2.60E-02	5.90E-02
										Fatty acid biosynthesis	6	3.80E-02	8.20E-02
										Phenylalanine metabolism	6	5.10E-02	1.10E-01
										Tyrosine metabolism	8	5.20E-02	1.10E-01
										Neuroactive ligand-receptor interaction	7	6.00E-02	1.20E-01
										Pantothenate and CoA biosynthesis	5	7.50E-02	1.50E-01
										Glycerolipid metabolism	8	7.80E-02	1.50E-01
										Ether lipid metabolism	5	1.00E-01	1.90E-01

Fig. S3. PQ upregulates genes involved in development and growth and down-regulates genes involved in degradation, metabolic pathways, and energy generation

This Table shows all the KEGG pathways of genes significantly up- and down- regulated by PQ, obtained using the online analysis tool DAVID (Database for Annotation, Visualization, and Integrated Discovery; Version 6.8) (*62, 63*). 19% of up-regulated and 9.5% of down-regulated genes fall into defined KEGG pathways. When these KEGG pathways are placed into broad categories of biological processes it can be seen that PQ up-regulates development, growth, and quality control, while down-regulating processes involved in degradation and metabolism. KEGG pathways are biochemical pathways defined by the Kyoto Encyclopedia of Genes and Genomes (*64*). Counts represent the number of genes in the KEGG pathway that were among the list of significantly up or down-regulated genes. P-values are based on Fisher's exact test to measure the gene-enrichment in the pathway. Padj represents adjusted p-values which control for false discovery rate with multiple comparisons using the method of (*83*). A shortened and simplified depiction of this analysis is presented in Fig. 2.

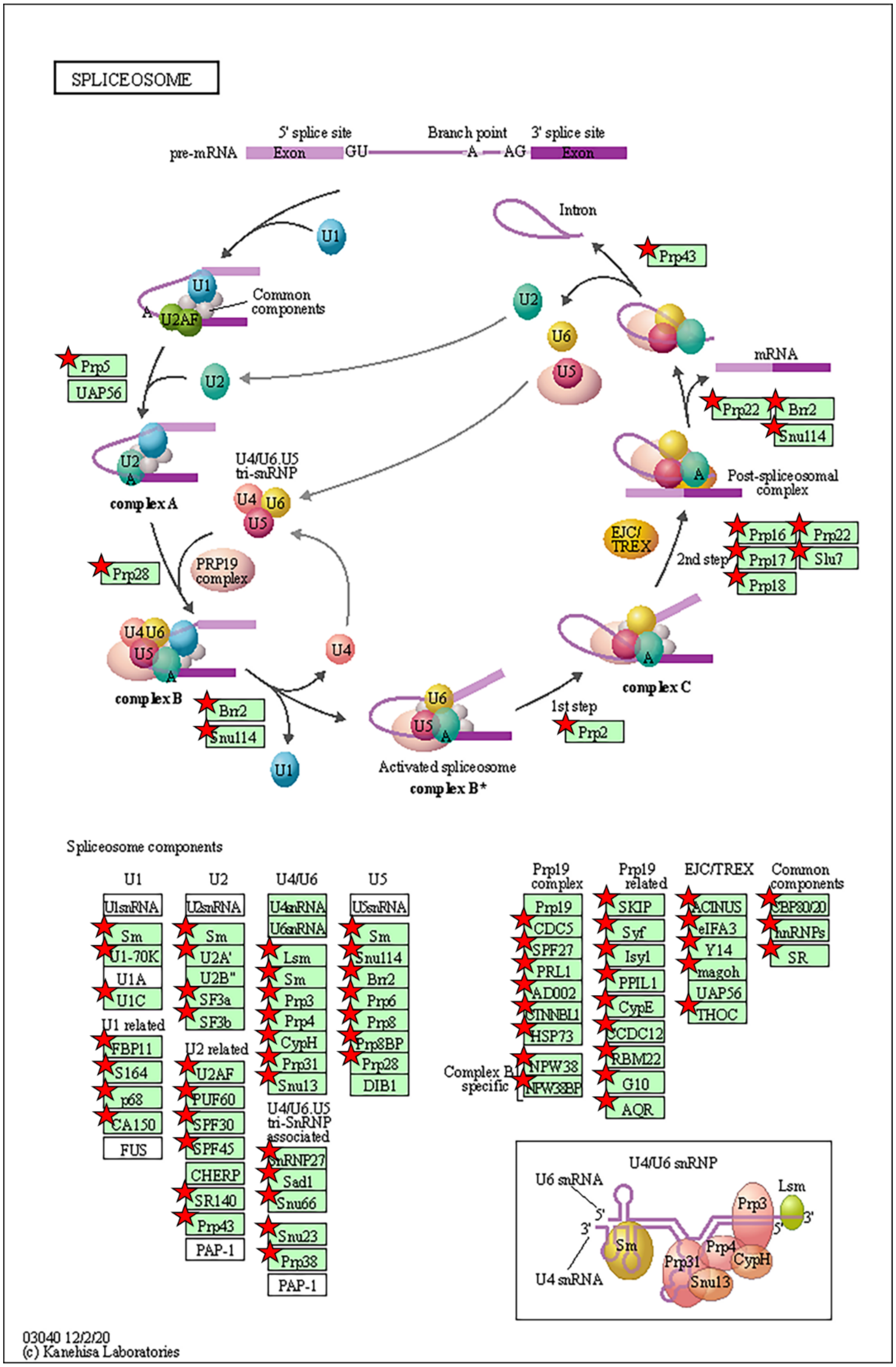

Fig. S4. The Spliceosome KEGG pathway
Proteins with homologs in *C. elegans* are shaded in green. Proteins which are encoded by genes that are significantly up-regulated by PQ treatment are indicated with a red star. Genes encoding 101/106 proteins in the pathway are up-regulated by PQ treatment. KEGG pathway cel03040 reproduced with permission (*64*).

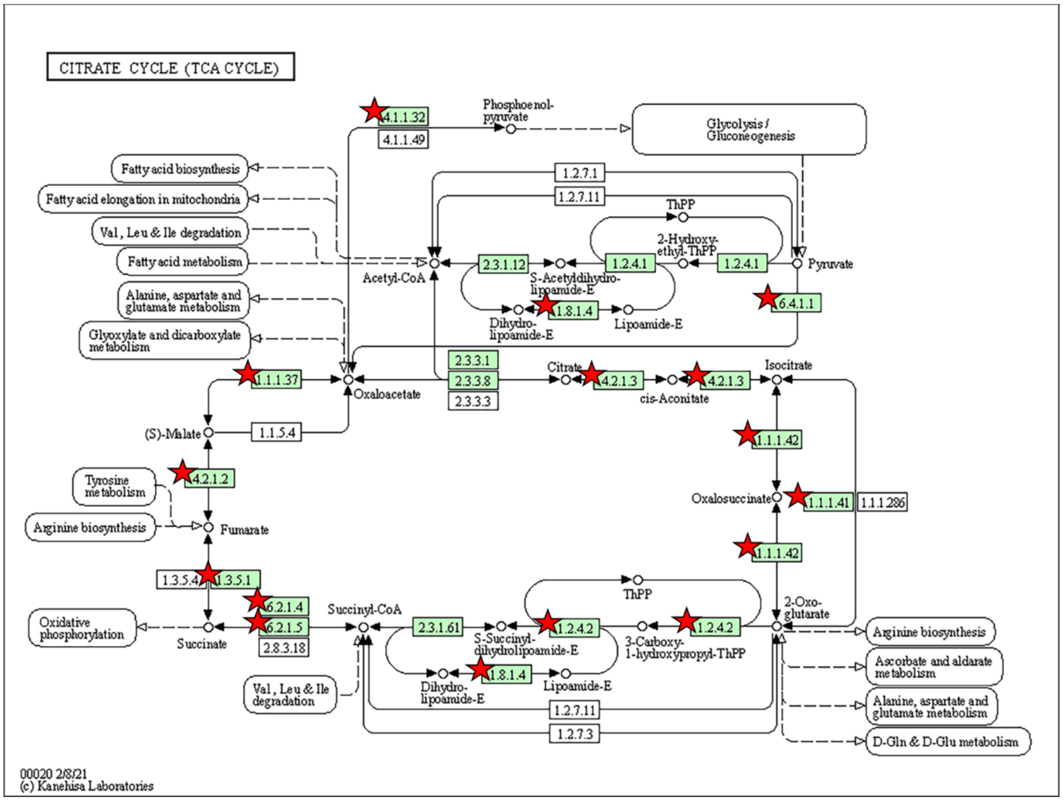

Fig. S5. The Citrate Cycle (TCA cycle) KEGG pathway

Enzymes with homologs in *C. elegans* are shaded in green. Enzymes which are encoded by genes that are significantly down-regulated by PQ treatment are indicated with a red star. Genes encoding 18/33 enzymes in the pathway are down-regulated by PQ treatment. KEGG pathway cel00020 reproduced with permission (*64*).

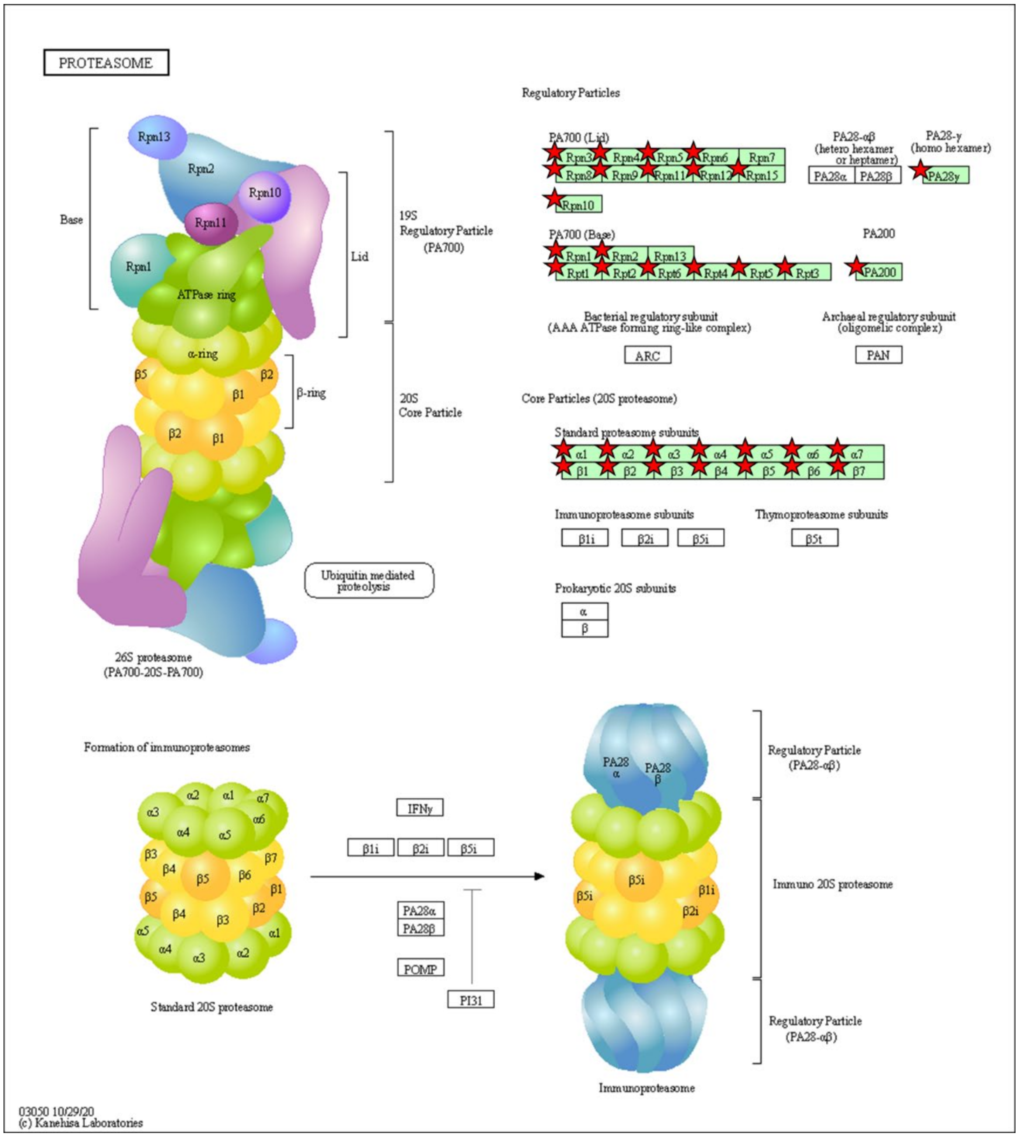

Fig. S6. The Proteasome KEGG pathway

Subunits with homologs in *C. elegans* are shaded in green. Subunits which are encoded by genes that are significantly up-regulated by PQ treatment are indicated with a red star. Genes encoding 35/38 subunits with homologues in *C. elegans* are up-regulated by PQ treatment. KEGG pathway cel03050 reproduced with permission (*64*).

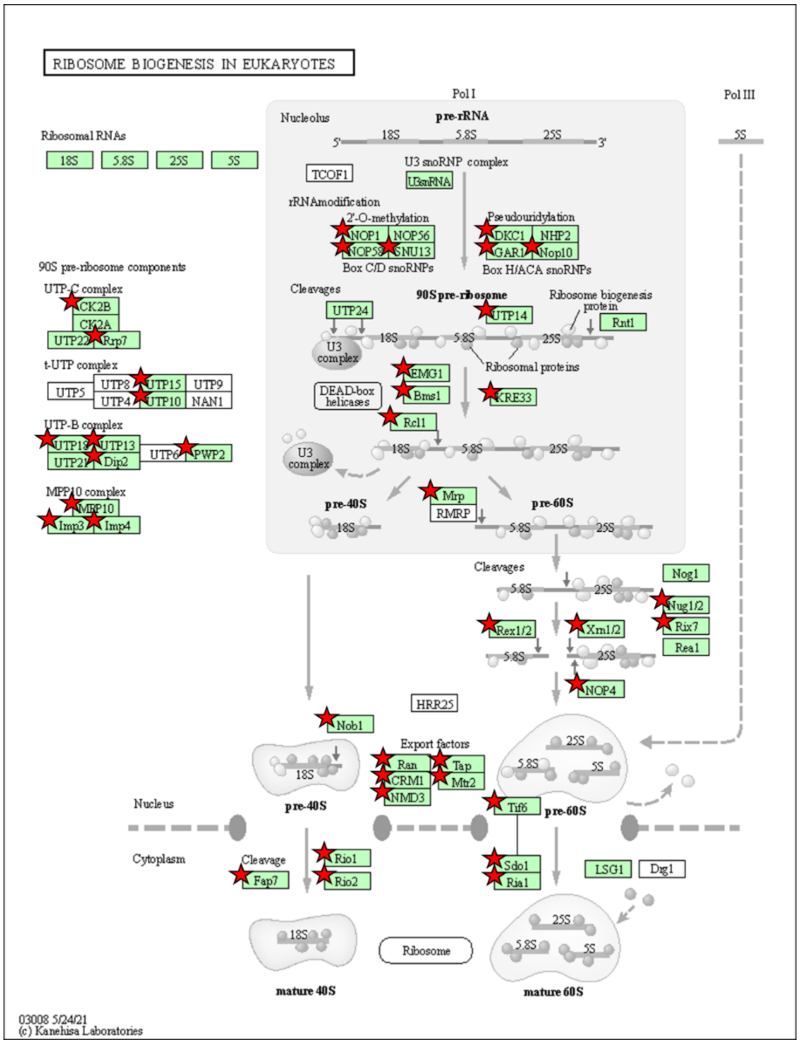

Fig. S7. Ribosome Biogenesis in Eukaryotes KEGG Pathway

Proteins with homologs in *C. elegans* are shaded in green. Proteins which are encoded by genes that are significantly up-regulated by PQ treatment are indicated with a red star. Genes encoding 49/78 proteins with subunits in *C. elegans* are up-regulated by PQ treatment. KEGG pathway cel03008 reproduced with permission (*64*).

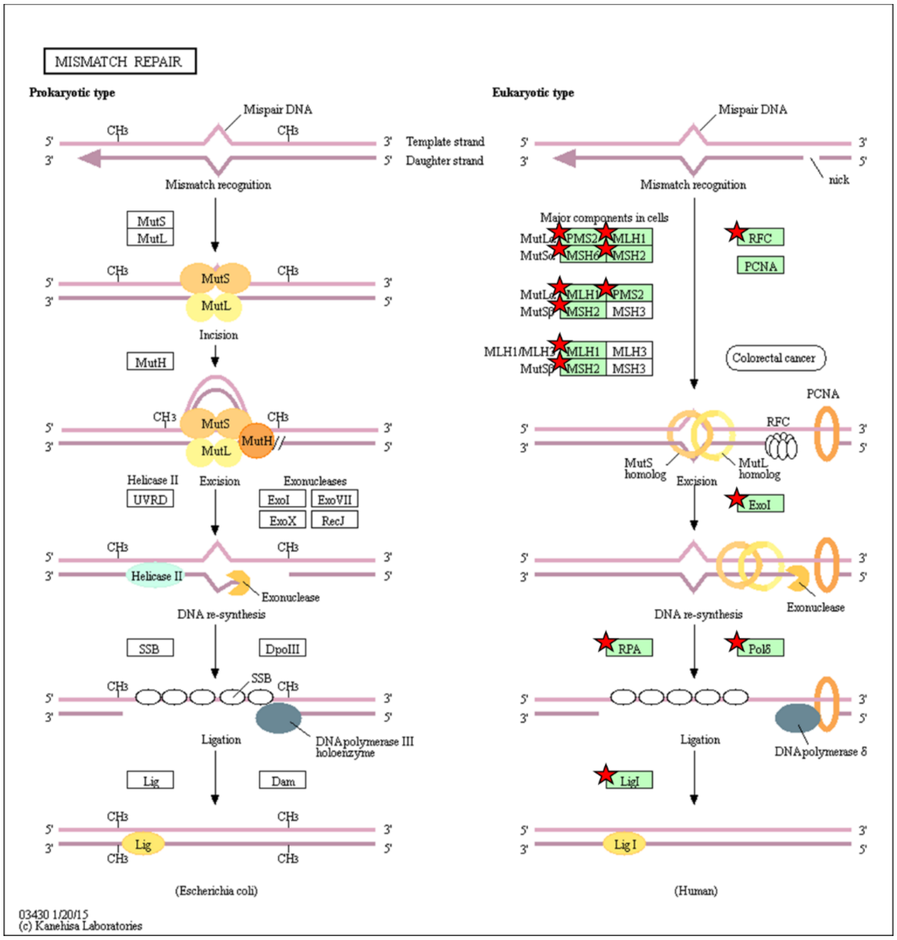

Fig. S8. Mismatch Repair KEGG Pathway

Proteins with homologs in *C. elegans* are shaded in green. Proteins which are encoded by genes that are significantly up-regulated by PQ treatment are indicated with a red star. Genes encoding 16/18 proteins with homologues in *C. elegans* are up-regulated by PQ treatment. KEGG pathway cel03430 reproduced with permission (*64*).

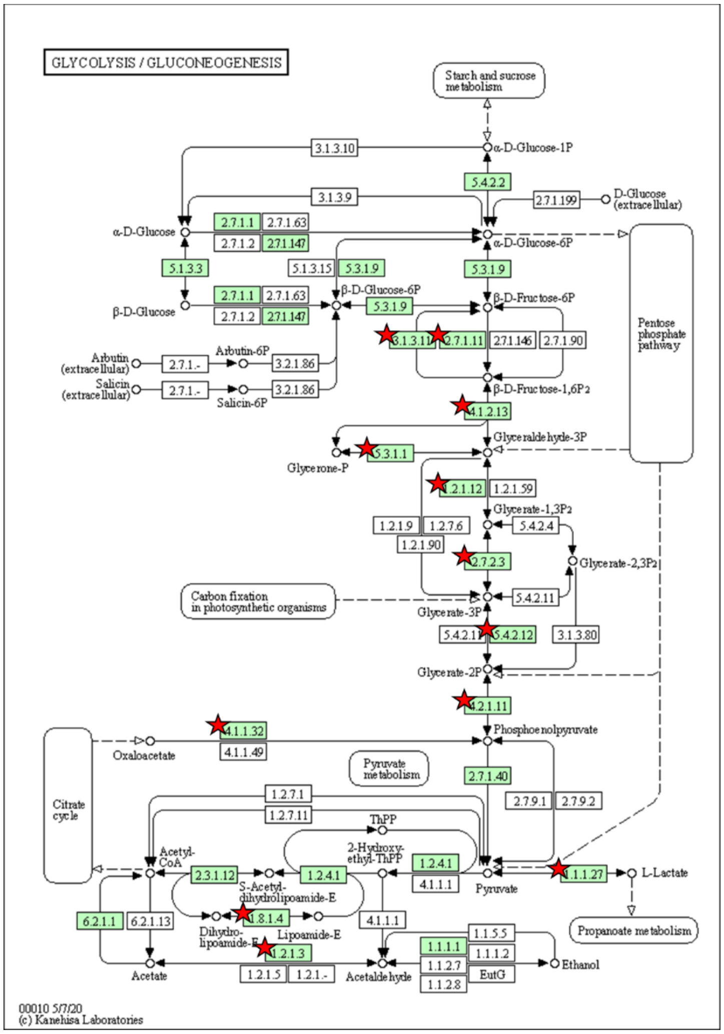

Fig. S9. Glycolysis/Gluconeogenesis KEGG pathway

Enzymes with homologs in *C. elegans* are shaded in green. Enzymes which are encoded by genes that are significantly down-regulated by PQ treatment are indicated with a red star. Genes encoding 16/39 enzymes with homologues in *C. elegans* are down-regulated by PQ treatment. KEGG pathway cel00010 reproduced with permission (*64*).

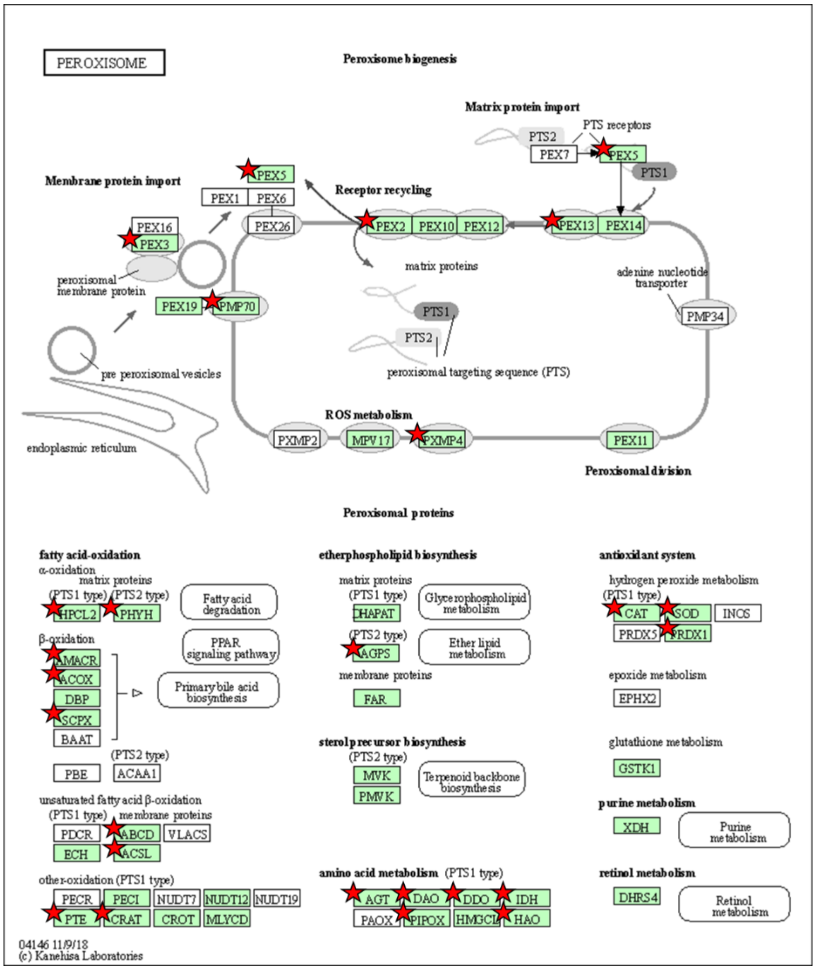

Fig. S10. Peroxisome KEGG pathway

Proteins with homologs in *C. elegans* are shaded in green. Proteins which are encoded by genes that are significantly down-regulated by PQ treatment are indicated with a red star. Genes encoding 30/63 proteins with homologues in *C. elegans* are down-regulated by PQ treatment. KEGG pathway cel04146 reproduced with permission (*64*).

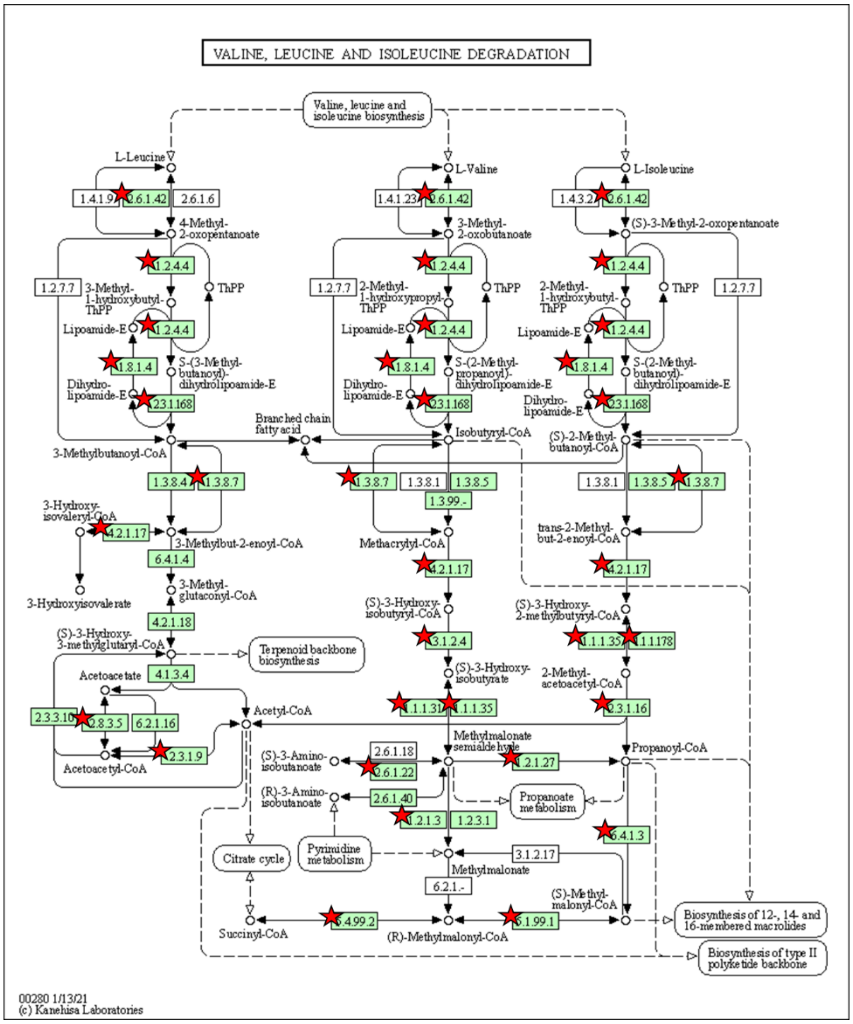

Fig. S11. Valine, Leucine, and Isoleucine Degradation KEGG pathway
Enzymes with homologs in *C. elegans* are shaded in green. Enzymes which are encoded by genes that are significantly down-regulated by PQ treatment are indicated with a red star. Genes encoding 33/40 enzymes with homologues in *C. elegans* are down-regulated by PQ treatment. KEGG pathway cel00280 reproduced with permission (*64*).

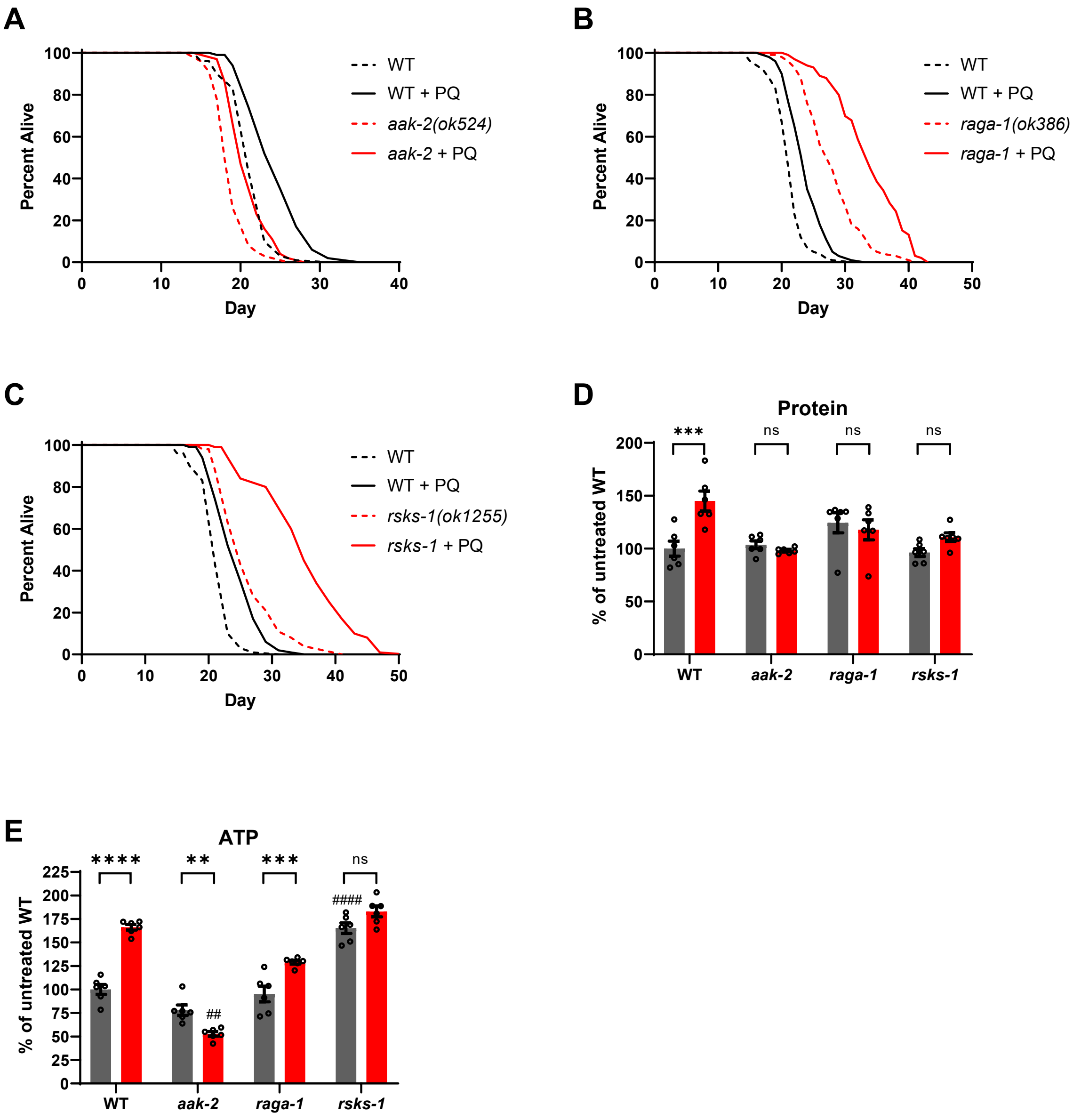

Fig. S12. PQ does not require the AMPK or TOR signaling pathways to increase lifespan but may involve these pathways to affect protein and ATP levels.
(**A**) Lifespan of *aak-2(ok524)* mutants treated with 0.1 mM PQ from hatching. (**B**) Lifespan of *raga-1(ok386)* mutants treated with 0.1 mM PQ from hatching. (**C**) Lifespan of *rsks-1(ok524)* mutants treated with 0.1 mM PQ from hatching. (**D**) Protein levels normalized to the mean level of untreated WT (58.60 ± 4.20 µg). (**E**) ATP levels normalized to the mean level of untreated WT (15.91 ± 0.84 pmol/µg protein). For (**A**-**C**) numerical values and statistics are presented in Table S1. For (**D**, **E**) bars represent means and error bars SEM, and the individual points are also plotted. Phenotypes of untreated and treated mutants are compared to untreated and treated WT, respectively, and significant differences are indicated above the bars with # (## $p<0.01$, #### $p<0.0001$). For each genotype, the untreated and treated phenotypes were also compared, and significant differences are indicated above the set of bars with asterisks (**$p<0.01$, ***$p<0.001$, ****$p<0.0001$).

	PQ up	PQ down	*daf-2* up	*daf-2* down	*eat-2* up	*eat-2* down	*glp-1* up	*glp-1* down	*rsks-1* up	*rsks-1* down	*nuo-6* up	*nuo-6* down	*isp-1* up	*isp-1* down	*pcca-1* up	*pcca-1* down	*metr-1* up	*metr-1* down
PQ up			3	22	2	19	6	40	2	32	6	22	5	7	33	2	36	2
PQ down			21	5	32	6	30	12	25	1	22	3	8	14	6	30	3	33
daf-2 up	3	21			11	8	18	5	21	2	33	1	23	2	8	12	6	14
daf-2 down	22	5			5	5	7	17	4	18	5	28	3	18	10	5	11	6
eat-2 up	2	32	11	5			17	12	14	1	13	4	6	14	2	29	2	30
eat-2 down	19	6	8	5			10	9	7	10	8	6	5	3	19	2	16	2
glp-1 up	6	30	18	7	17	10			32	3	21	7	8	5	11	23	9	27
glp-1 down	40	12	5	17	12	9			4	30	4	18	4	14	25	15	28	13
rsks-1 up	2	25	21	4	14	7	32	4			20	3	11	5	8	24	5	28
rsks-1 down	32	1	2	18	1	10	3	30			4	16	4	4	23	2	27	1
nuo-6 up	6	22	33	5	13	8	21	4	20	4			38	0	11	12	7	14
nuo-6 down	22	3	1	28	4	6	7	18	3	16			0	26	9	5	11	4
isp-1 up	5	8	23	3	6	5	8	4	11	4	38	0			6	6	6	6
isp-1 down	7	14	2	18	14	3	5	14	5	4	0	26			3	15	4	13
pcca-1 up	33	6	8	10	2	19	11	25	8	23	11	9	6	3			6	6
pcca-1 down	2	30	12	5	29	2	23	15	24	2	12	5	6	15			3	15
metr-1 up	36	3	6	11	2	16	9	28	5	27	7	11	6	4	6	3		
metr-1 down	2	33	14	6	30	2	27	13	28	1	14	4	6	13	6	15		

Fig. S13. Comparison of gene expression changes brought about by treatment of the WT with PQ and in long-lived mutants

One measure of how similar gene sets A and B are to each other is the percentage of genes that are common out of the total number of genes changed in either of the two sets (A U B). These percentages are what are shown in the table, which compares the genes sets changed by PQ treatment to the genes sets changed by various mutations as well as comparing the genes sets changed by the mutations to each other. When the percentage of genes changed is in the same direction (up or down) in the two sets being compared is 2- or more times greater than the percentage changed in the opposite direction the cell is shaded in pink. When the percentage of genes changed in the opposite direction in the two sets being compared is two or more times greater than the percentage changed in the same direction the cell is shaded in blue. The cells are shaded in yellow when the difference between the percentages corresponding to the two types of comparisons is not two-fold or greater. As shown in Fig. 6, when compared to the classical aging genes *daf-2, eat-2, glp-1, rsks-1* and *nuo-6*, PQ treatment tends to change many more genes in the opposite direction than in the same directions (blue cells rather than pink cells). In contrast, *pcca-1* and *metr-1* gene expression tends to be changed in the same direction (pink cells). There is no clear pattern for *isp-1*. As expected, comparison between *pcca-1* and *metr-1* and the aging genes partially reflects the similarity between their pattern of expression and that of PQ treatment: a preponderance of blue cells. However, when we only consider comparisons between gene expression changes among the long-lived mutants (in the box outlined in thick black lines), gene expression changes for genes in overlaps are almost entirely in the same direction, except for *isp-1* compared to *eat-2*, which shows no clear pattern.

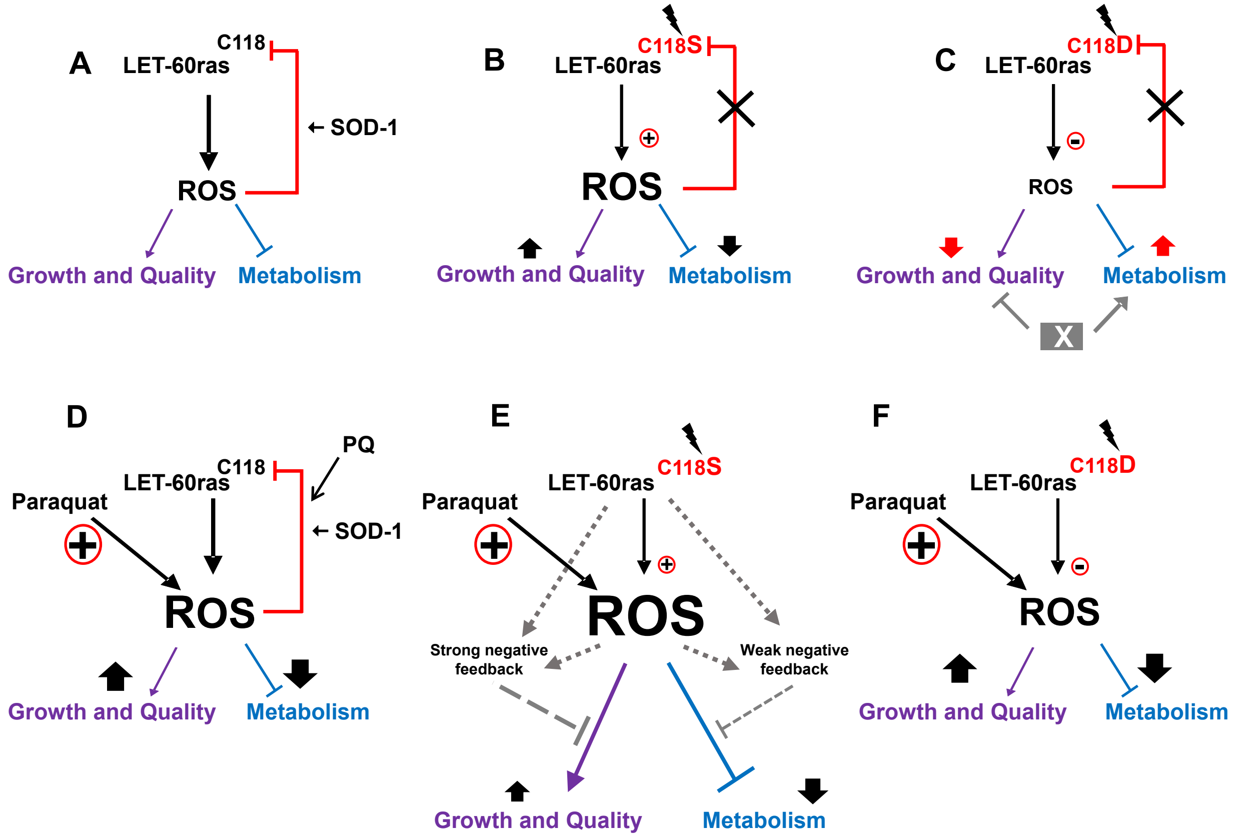

Fig. S14. A model of how RDRS affects gene expression

This figure is an elaboration of the model shown in Fig.7, depicting the situation in the WT, as well as in C118S and C118D mutants, in the absence and presence of PQ treatment. (**A**) The situation in the WT. LET-60ras signaling leads, directly or indirectly, to the production of ROS, hereafter referred to as RAS-dependent ROS signaling (RDRS), which affects gene expression. RDRS promotes the expression of genes associated with growth and the high-quality synthesis of cellular constituents (referred to as Growth and Quality) and inhibits the expression of genes necessary for intermediary metabolism and energy generation (referred to as Metabolism). Signaling through the RAS pathway is negatively regulated by oxidation of cysteine C118 of LET-60ras. This effect, which requires SOD-1, appears to act as negative feedback from RDRS (red arrow). (**B**) The situation in C118S mutants. C118S cannot be oxidized by ROS. In the absence of negative feedback from RDRS, RAS signaling is increased, leading to an increase in RDRS and thus, to an up-regulation of genes associated with Growth and Quality, and a down-regulation of genes necessary for Metabolism. (**C**) The situation in C118D mutants. C118D mimics C118 oxidation and is likely resistant or insensitive to feedback regulation. However, as it mimics oxidized C118 it leads to a downregulation of RAS signaling and a decrease of RDRS. This reveals the existence of an opposing signaling pathway (X) that affects global gene expression but acts in the opposite direction from RAS. In the absence of strong RDRS, the unknown X pathway dominates, which results in a down-regulation of genes associated with Growth and Quality, and an up-regulation of genes necessary for Metabolism. **(D**) The situation in the WT treated with PQ. ROS generated by PQ acts downstream of LET-60ras to enhance RDRS resulting in an up-regulation of genes associated with Growth and Quality, and a down-regulation of genes necessary for Metabolism. RDRS is increased to a higher degree than in C118S mutants, despite a possible increase in the negative feedback through C118. As described, PQ in a SOD-1-dependant manner, is also capable of oxidizing C118. However, this does not affect the stimulation of RDRS by PQ, given that it acts downstream of LET-60ras. (**E**) The situation in C118S mutants treated with PQ. The absence of the normal feedback provided by oxidation of C118 leads to chronic activation of LET-60ras. This activates a signal-dampening mechanism, which are well-known to exist downstream of RAS signaling. We expect this mechanism to be a normal part of RDRS, but its importance is most obviously revealed in C118S mutants treated with PQ, which is why it is only shown for this situation. When C118S mutants are treated with PQ, the dampening mechanism prevents the

artificial elevation of ROS by PQ to have major effects on gene expression in C118S mutants. The dampening of the effect of PQ in the mutants appears to be more pronounced for up-regulated genes than for down-regulated genes. (**F**) The situation in C118D mutants treated with PQ. As discussed above, RAS signaling is down-regulated in C118D mutants, but because it acts downstream of LET-60, PQ can nonetheless enhance RDRS. Thus, the weakened LET-60ras signal is bypassed with an intense downstream ROS signal, which overcomes the effect of X and produces a pattern of gene expression similar to that of the WT treated with PQ.

Table S1. Numerical values and statistics for aging data presented in Fig. 1 and Fig. S12.

Genotype	Sample size N (repeats)	Mean Lifespan ± SEM	P value (significance) [a,b]
WT	150 (3)	20.45 ± 0.31	
sod-1(tm783)	150 (3)	17.87 ± 0.34	WT: 0.0001(****)
sod-2(ok1030)	150 (3)	28.43 ± 0.47	WT: 0.0001(****)
sod-1; sod-2	100 (2)	17.82 ± 0.40	*sod-1(tm783)*: 0.999 (ns)
			sod-2(ok1030): 0.0001(****)
WT	150 (3)	20.45 ± 0.31	
sod-1(tm783)	150 (3)	17.87 ± 0.34	WT: 0.0001 (****)
ctl-1(ok1242)	150 (3)	19.81 ± 0.37	WT: 0.8597 (ns)
ctl-2(ok1137)	150 (3)	19.64 ± 0.35	WT: 0.5986 (ns)
ctl-3(ok2042)	150 (3)	20.08 ± 0.35	WT: 0.9965 (ns)
sod-1 ctl-1	100 (2)	20.06 ± 0.46	WT: 0.9979 (ns)
			sod-1(tm783): 0.0001 (****)
sod-1 ctl-2	100(2)	19.34 ± 0.37	WT: 0.30947 (ns)
			sod-1(tm783): 0.0130 (*)
sod-1 ctl-3	100(2)	18.54 ± 0.33	WT: 0.0033 (**)
			sod-1(tm783): 0.4382 (ns)
WT	150 (3)	20.45 ± 0.31	
WT + 0.1mM PQ	150 (3)	25.01 ± 0.44	WT: 0.0001(****)
sod-1(tm783)	150 (3)	17.87 ± 0.34	
sod-1(tm783) + 0.1mM PQ	150 (3)	19.17 ± 0.36	*sod-1*: 0.07 (ns)
sod-1(tm783)	150 (3)	17.32 ± 0.22	
sod-1(tm783) + 0.1mM PQ	100 (2)	22.54 ± 0.47	*sod-3*: 0.0001(****)
sod-4(gk101)	150 (3)	19.29 ± 0.33	
sod-4(gk101) + 0.1mM PQ	150 (3)	24.43 ± 0.44	*sod-4*: 0.0001(****)
sod-5(tm1146)	150 (3)	19.73 ± 0.32	
sod-5(tm1146) + 0.1mM PQ	150 (3)	25.23 ± 0.47	*sod-5*: 0.0001(****)
WT	100 (2)	19.14 ± 0.33	
WT + 0.1mM PQ	100 (2)	26.74 ± 0.56	WT: 0.0001(****)
let-60–C118S	100 (2)	18.86 ± 0.29	WT: 0.9778(ns)

let-60–C118S + 0.1mM PQ	100 (2)	22.16 ± 0.32	WT + 0.1mM PQ: 0.0001(****)<br>*let-60*–C118S: 0.0001(****)
WT	100 (2)	17.87 ± 0.26	
WT + 0.1mM PQ	100 (2)	25.66 ± 0.39	WT: 0.0001(****)
let-60–C118D	100 (2)	19.06 ± 0.32	WT: 0.0512(ns)
let-60–C118D + 0.1mM PQ	100 (2)	25.57 ± 0.42	WT + 0.1mM PQ: 0.9996 (ns)<br>*let-60*–C118D: 0.0001(****)
WT	200 (4)	19.18 ± 0.14	
nuo-6(qm200)	200 (4)	37.89 ± 0.45	WT: 0.0001(****)
let-60–C118S	200 (4)	18.92 ± 0.14	WT: 0.19(ns)
nuo-6; let-60–C118S	200 (4)	21.42 ± 0.21	WT: 0.0001(****)<br>*nuo-6*: 0.0001(****)
WT	100 (2)	21.54 ± 0.26	
WT + 0.1mM PQ	100 (2)	24.60 ± 0.35	WT: 0.0001(****)
aak-2(ok524)	100 (2)	18.74 ± 0.20	WT: 0.0001(****)
aak-2(ok524) + 0.1mM PQ	100 (2)	20.86 ± 0.25	*aak-2*: 0.0001(****)
WT	100 (2)	21.24 ± 0.26	
WT + 0.1mM PQ	100 (2)	23.80 ± 0.29	WT: 0.0001(****)
raga-1(ok386)	100 (2)	28.00 ± 0.45	WT: 0.0001(****)
raga-1(ok386) + 0.1mM PQ	100 (2)	33.71 ± 0.53	*raga-1*: 0.0001(****)
WT	100 (2)	21.54 ± 0.26	
WT + 0.1mM PQ	100 (2)	24.60 ± 0.35	WT: 0.0001(****)
rsks-1(ok1255)	100 (2)	25.99 ± 0.42	WT: 0.0001(****)
rsks-1(ok1255) + 0.1mM PQ	100 (2)	35.20 ± 0.69	*rsks-1*: 0.0001(****)

[a]P-values are from One-way Anova. Significance thresholds are adjusted for multiple comparisons using the Šídák method.
[b]The control to which the experimental strain/condition is compared is indicated.

Table S2. Gene Ontology Cellular Component (GO_CC) Terms for genes up-regulated by PQ

Cellular component (CC) Gene Ontology (GO) terms of genes Up-regulated by PQ	Count	P-Value	Benjamini
nucleus	1014	1.30E-144	8.30E-142
cytoplasm	743	3.80E-75	1.20E-72
nucleolus	88	3.30E-30	7.20E-28
cell cortex	67	9.50E-23	1.50E-20
chromosome	72	2.10E-22	2.80E-20
cytosol	165	4.60E-21	5.00E-19
nucleoplasm	46	1.00E-20	9.40E-19
P granule	54	3.20E-20	2.60E-18
mitochondrion	207	1.10E-19	8.10E-18
proteasome complex	35	6.80E-17	4.40E-15
spliceosomal complex	37	4.00E-16	2.40E-14
condensed chromosome	33	7.60E-16	4.10E-14
catalytic step 2 spliceosome	36	1.40E-15	7.00E-14
kinetochore	34	1.40E-14	6.40E-13
condensed nuclear chromosome	31	7.00E-14	3.00E-12
nuclear pore	30	1.40E-12	5.50E-11
cytoplasmic mRNA processing body	31	2.20E-12	8.40E-11
chromosome, centromeric region	24	4.10E-12	1.50E-10
Golgi apparatus	83	5.50E-12	1.90E-10
condensed chromosome kinetochore	23	1.40E-11	4.60E-10
perinuclear region of cytoplasm	50	2.70E-11	8.30E-10
precatalytic spliceosome	23	1.20E-10	3.60E-09
spindle	26	1.30E-10	3.60E-09
mitochondrial large ribosomal subunit	21	1.60E-10	4.40E-09
small-subunit processome	28	2.50E-10	6.40E-09
centrosome	39	3.90E-10	9.70E-09
endoplasmic reticulum	110	6.30E-10	1.50E-08
microtubule organizing center	22	2.40E-09	5.40E-08
endoplasmic reticulum membrane	77	2.40E-09	5.40E-08
nuclear envelope	33	3.10E-09	6.70E-08
Golgi membrane	58	1.10E-08	2.40E-07
U4/U6 x U5 tri-snRNP complex	17	2.10E-08	4.20E-07
mitochondrial small ribosomal subunit	21	3.20E-08	6.40E-07
DNA-directed RNA polymerase III complex	16	7.00E-08	1.30E-06
U2 snRNP	17	1.40E-07	2.60E-06
U1 snRNP	16	4.30E-07	7.90E-06
mediator complex	17	6.40E-07	1.10E-05
U2-type prespliceosome	14	7.70E-07	1.20E-05
proteasome core complex	14	7.70E-07	1.20E-05
DNA-directed RNA polymerase II, core complex	14	7.70E-07	1.20E-05
nuclear exosome (RNase complex)	13	2.50E-06	4.00E-05
endosome	26	3.50E-06	5.40E-05
microtubule	39	4.20E-06	6.40E-05
cleavage furrow	15	5.70E-06	8.40E-05
preribosome, large subunit precursor	20	6.90E-06	1.00E-04
intracellular ribonucleoprotein complex	67	9.10E-06	1.30E-04
spindle midzone	13	1.30E-05	1.80E-04
integral component of endoplasmic reticulum membrane	21	1.40E-05	1.90E-04
nuclear chromosome	14	1.70E-05	2.20E-04
spindle pole	14	1.70E-05	2.20E-04
collagen trimer	76	1.90E-05	2.40E-04
nuclear membrane	16	2.00E-05	2.50E-04
transcription factor TFIID complex	11	2.70E-05	3.30E-04
mitotic spindle	12	4.00E-05	4.60E-04
proteasome regulatory particle, base subcomplex	12	4.00E-05	4.60E-04
U5 snRNP	12	4.00E-05	4.60E-04
ribosome	69	4.80E-05	5.50E-04
cytoplasmic exosome (RNase complex)	10	8.70E-05	9.40E-04
nuclear periphery	10	8.70E-05	9.40E-04
spindle microtubule	10	8.70E-05	9.40E-04
eukaryotic 43S preinitiation complex	11	1.20E-04	1.30E-03
cytoplasmic vesicle	30	1.70E-04	1.80E-03
germ cell nucleus	9	2.80E-04	2.80E-03
centriole	9	2.80E-04	2.80E-03
midbody	10	3.60E-04	3.50E-03
DNA-directed RNA polymerase I complex	10	3.60E-04	3.50E-03
eukaryotic 48S preinitiation complex	11	3.90E-04	3.80E-03
Prp19 complex	8	8.80E-04	8.00E-03
U6 snRNP	8	8.80E-04	8.00E-03
core mediator complex	8	8.80E-04	8.00E-03
COP9 signalosome	8	8.80E-04	8.00E-03
eukaryotic translation initiation factor 3 complex	12	9.10E-04	8.30E-03
dynactin complex	9	1.00E-03	9.10E-03
cytoplasmic side of plasma membrane	9	1.00E-03	9.10E-03
nuclear chromosome, telomeric region	10	1.10E-03	9.20E-03
ubiquitin ligase complex	13	1.60E-03	1.40E-02
endosome membrane	12	1.90E-03	1.60E-02
SCF ubiquitin ligase complex	10	2.60E-03	2.00E-02
phagocytic vesicle	10	2.60E-03	2.00E-02
early endosome	16	2.70E-03	2.00E-02
cortical granule	7	2.70E-03	2.00E-02
prefoldin complex	7	2.70E-03	2.00E-02
proteasome core complex, alpha-subunit complex	7	2.70E-03	2.00E-02
Golgi transport complex	7	2.70E-03	2.00E-02
metaphase plate	7	2.70E-03	2.00E-02
replication fork protection complex	7	2.70E-03	2.00E-02
spliceosomal tri-snRNP complex	7	2.70E-03	2.00E-02
exosome (RNase complex)	7	2.70E-03	2.00E-02
cyclin-dependent protein kinase holoenzyme complex	8	3.00E-03	2.10E-02
late endosome membrane	8	3.00E-03	2.10E-02
mitochondrial ribosome	8	3.00E-03	2.10E-02
nuclear proteasome complex	8	3.00E-03	2.10E-02
intracellular	136	4.20E-03	2.90E-02

Table S2 shows the GO CC terms of genes significantly up-regulated by PQ, obtained using the online analysis tool DAVID (Database for Annotation, Visualization, and Integrated Discovery; version 6.8) (*62, 63*). 3222 (59.0%) of the up-regulated genes are represented by these terms. However, in the Table only GO CC terms with a Padj < 0.05 are shown. P-values are based on Fisher's exact test to measure the gene-enrichment in the pathway. Padj represents adjusted p-values which control for false discovery rate with multiple comparisons by using the linear step-up method of (*83*).

Table S3. Gene Ontology Cellular Component Terms (GO_CC) for genes down-regulated by PQ

**Cellular component (CC) Gene Ontology (GO) terms of genes Down-regulated by PQ**	**Count**	**P-Value**	**Benjamini**
extracellular space	142	7.00E-26	2.20E-23
pseudopodium	36	9.70E-18	1.50E-15
extrinsic component of cytoplasmic side of plasma membrane	32	1.60E-10	1.70E-08
intracellular membrane-bounded organelle	60	7.10E-10	5.50E-08
cytoskeleton	98	9.20E-10	5.70E-08
membrane raft	42	3.80E-09	2.00E-07
extracellular region	129	4.80E-09	2.10E-07
peroxisome	25	4.80E-07	1.90E-05
basement membrane	15	8.80E-06	2.90E-04
M band	19	9.30E-06	2.90E-04
striated muscle dense body	43	1.40E-05	4.00E-04
lysosome	33	3.00E-04	7.80E-03
myofibril	10	3.30E-04	7.80E-03
chloride channel complex	18	4.60E-04	1.00E-02
proteinaceous extracellular matrix	17	1.60E-03	3.20E-02

Table S3 shows all the GO CC terms of genes significantly down-regulated by PQ, obtained using the online analysis tool DAVID (Database for Annotation, Visualization, and Integrated Discovery; version 6.8) (*62, 63*). 2788 (56.9%) of the down-regulated genes are represented by these terms, However, in the Table only GO CC terms with a Padj < 0.05 are shown. P-values are based on Fisher's exact test to measure the gene-enrichment in the pathway. Padj represents adjusted p-values which control for false discovery rate with multiple comparisons by using the linear step-up method of (*83*).

Other Supplementary Materials for this manuscript includes the following file:

Data S1: Significantly Changed Genes.xlsx

This Excel file contains the names of the genes that were significantly up-regulated and down-regulated in the RNAseq analyses carried out in this study.
